# Antioxidative Effects of Defatted Rice Bran in Rats on AOM/DSS‐Induced Colon Oxidative Stress and Correlations Between Gut Microbiota and Antioxidant Biomarkers

**DOI:** 10.1002/fsn3.70554

**Published:** 2025-07-29

**Authors:** Kansuda Wunjuntuk, Laleewan Tajasuwan, Aikkarach Kettawan, Thanaporn Rungruang, Pinidphon Prombutara, Pattaneeya Prangthip, Akkarapol Chaisri, Nilesh Nirmal, Aurawan Kringkasemsee Kettawan

**Affiliations:** ^1^ Department of Home Economics Faculty of Agriculture, Kasetsart University Bangkok Thailand; ^2^ Department of Tropical Nutrition and Food Science Faculty of Tropical Medicine, Mahidol University Bangkok Thailand; ^3^ Institute of Nutrition, Mahidol University Nakhon Pathom Thailand; ^4^ Department of Anatomy Faculty of Medicine, Siriraj Hospital, Mahidol University Bangkok Thailand; ^5^ Mod Gut Co., Ltd Bangkok Thailand; ^6^ Omics Science and Bioinformatics Centre Faculty of Science, Chulalongkorn University Bangkok Thailand

**Keywords:** antioxidants, colorectal cancer, defatted rice bran, gut microbiota, oxidative stress

## Abstract

Defatted rice bran (DRB), a by‐product of Thai *Oryza sativa* , is rich in dietary fiber and antioxidant phytochemicals. This study investigated the bioaccessibility, antioxidative efficacy, and prebiotic potential of DRB in colorectal cancer (CRC) models. Simulated gastrointestinal digestion demonstrated high bioaccessibility of flavonoids (79%) and phenolics (62%), along with substantial antioxidant activity: DPPH (57%), FRAP (83%), and ORAC (74%). In vitro, digested DRB significantly reduced intracellular reactive oxygen species (ROS; −33%, *p* < 0.05) and suppressed IL‐6, IL‐8, and TNF‐α production in Caco‐2 cells. In vivo, male Wistar rats (*n* = 5/group) were exposed to azoxymethane/dextran sulfate sodium (AOM/DSS) and supplemented with DRB (3 or 6 g/kg body weight/day) for 90 days. High‐dose DRB markedly reduced protein carbonyls (−71%) and 8‐OHdG (−29%), while restoring glutathione (+26%), superoxide dismutase (+125%), and catalase (+50%) relative to AOM/DSS controls (*p* < 0.05). 16S rRNA sequencing revealed a reduction in Proteobacteria (−58%) and an increased abundance of *Akkermansia*, *Lactobacillus*, and a genus belonging to Ruminococcaceae, which positively correlated with short‐chain fatty acids and antioxidant biomarkers (*r* > 0.6, FDR < 0.05). These findings indicate that DRB possesses potent antioxidative and prebiotic properties, supporting its use as a safe, multifunctional dietary ingredient for CRC risk reduction.

## Graphical abstract

Defatted rice bran (DRB), a by‐product of Thai rice oil extraction, demonstrated significant antioxidant and prebiotic properties in AOM/DSS‐induced colorectal cancer models. DRB supplementation reduced oxidative stress markers, enhanced antioxidant enzymes, and favorably modulated gut microbiota, particularly increasing *Akkermansia*, *Lactobacillus* and a genus belonging to Ruminococcaceae, which correlated with improved SCFA production and redox balance. These findings support DRB as a multifunctional dietary ingredient for colorectal cancer prevention.

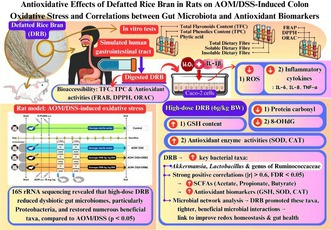

**Abbreviations**
8‐OHdG: 8‐hydroxy‐2′‐deoxyguanosine
AOM: azoxymethane
BW: body weight
CAT: catalase
CRC: colorectal cancer
DPPH: 2,2‐diphenyl‐1‐picrylhydrazyl
DRB: defatted rice bran
DSS: dextran sulfate sodium
FRAP: ferric reducing antioxidant power
GAE: gallic acid equivalents
GPx: glutathione peroxidase
GSH: glutathione
IBD: inflammatory bowel disease
IL‐6: interleukin 6
IL‐8: interleukin 8
ORAC: oxygen radical antioxidant capacity
QE: quercetin equivalent
ROS: reactive oxygen species
SOD: superoxide dismutase
TE: trolox equivalents
TFC: total flavonoid content
TNF‐α: tumor necrosis factor‐alpha
TPC: total phenolic content

## 1 Introduction

Colorectal cancer (CRC) is the second leading cause of cancer‐related deaths globally. Its complex pathogenesis involves inflammation and oxidative stress, which can oxidize biomolecules or activate inflammatory pathways, influencing tumor initiation and cancer cell survival. Conditions like inflammatory bowel disease (IBD) increase CRC risk, with chronic inflammation and oxidative stress as significant contributors (Bardelčíková et al. 2023). Literature suggests that natural bioactive compounds are safe alternatives to treat CRC conditions due to their low toxicity and fewer side effects compared to synthetic drugs (Huang et al. 2019). For example, Deng et al. (2024) reported that curcumin extracted from turmeric suppressed tumor formation by restoring the gut microbiota in an AOM/DSS‐induced CRC model mice.

Additionally, curcumin can improve colon length and structural morphology (Deng et al. 2024). Feeding rats with 8 mg/kg resveratrol from grapes for 15 weeks can significantly decrease ROS production, attenuate DNA damage levels, suppress lipid and protein oxidation, and downregulate JNK and c‐JUN in colon tissue, which can exert strong antioxidative and anti‐inflammatory effects in rats with 1,2‐dimethylhydrazine (DMH)‐induced CRC (Maleki et al. 2024). Cruciferous vegetables, such as broccoli, Brussels sprouts, cauliflower, cabbage, and kale, are rich in nutrients and a good source of dietary fiber, which has the potential to prevent tumor formation and reduce CRC incidence (Ağagündüz et al. 2022). Natural diets and functional food ingredients have garnered increasing interest as sustainable, accessible approaches for reducing the risk of non‐communicable diseases, including colorectal cancer (CRC). Public awareness of preventive health through dietary modulation is expanding globally, with a particular focus on gut health and oxidative stress balance.

Thailand, one of the world's largest rice producers, generates substantial quantities of rice bran as a by‐product of rice milling. A solvent extraction method was used to extract a combination of Thai rice bran varieties ( *Oryza sativa* ) into rice bran oil. Defatted rice bran (DRB), the residual material after this process, is frequently underutilized or diverted to animal feed. However, several studies have found that DRB contains valuable bioactive compounds, such as phenolic acids, flavonoids, and dietary fibers, as well as essential vitamins and minerals. DRB is recognized for its substantial dietary fiber content, which ranges from 24% to 32% and includes both soluble and insoluble fibers. Furthermore, DRB is a valuable source of plant‐based proteins (16%–22%) and essential nutrients such as vitamin B1, vitamin B3, folate, phosphorus, calcium, potassium, magnesium, iron, and zinc (Mukprasirt et al. 2023; Tajasuwan et al. 2022). In addition, DRB is rich in phenolic acids such as ferulic, *p*‐coumaric, vanillic, syringic, and caffeic acids, which exhibit potent antioxidant properties. The structure and radical‐neutralizing capacity of phenolic acids are influenced by the type and position of substituents on the aromatic ring (Chen et al. 2020; Platzer et al. 2022). Tricin is the main flavonoid in rice bran, followed by luteolin, apigenin, and quercetin (Goufo and Trindade 2014). They offer health benefits, including antioxidant effects that may lower the risk of cancer, cardiovascular diseases, and neurodegenerative disorders (Jiang et al. 2020; Prasher et al. 2022). These phytoactive compounds use various mechanisms of electron transfer to neutralize free radicals, chelate metal ions, inhibit oxidase enzymes, and activate antioxidant enzymes while modulating cellular signaling pathways (Boadi et al. 2021; Lin et al. 2015). Additionally, DRB contains a significant amount of phytic acid, also referred to as inositol hexaphosphate or IP6. Phytic acid functions as an antioxidant by binding to metal ions and inhibiting oxidative reactions in the human body, thereby protecting cells and tissues (Lee et al. 2015; Pujol et al. 2023). Our previous studies reported that DRB reduces inflammation by downregulating proinflammatory cytokines through the NF‐kB signaling pathway, inhibits cancer cell proliferation, and decreases CRC tumorigenesis (Tajasuwan et al. 2022). Additionally, DRB acts as a prebiotic, promoting gut microbiota balance, restoring goblet cells, enhancing colonic mucus thickness, and increasing beneficial bacteria such as *Alloprevotella, Prevotellaceae UCG‐001, Ruminococcus, Roseburia*, and *Butyricicoccus* (Davani‐Davari et al. 2019). It also boosts short‐chain fatty acid (SCFA) production, particularly acetic acid, butyric acid, and propionic acid (Tajasuwan et al. 2023), which help reduce oxidative stress, modulate cytokine levels, and alleviate chronic inflammation (González‐Bosch et al. 2021; Zhang et al. 2022). There is strong evidence that SCFAs, particularly butyric acid, scavenge free radicals, inhibit lipid peroxidation (Jahns et al. 2015), enhance antioxidant‐enzyme production, and reduce ROS in colon cells (Zhou et al. 2023). Notwithstanding these discoveries, the precise influence of DRB on CRC‐related oxidative stress and host–microbiota–redox interactions remains inadequately investigated. This gap is particularly relevant in the context of rising interest in sustainable nutrition and functional foods, where the identification and revalorization of nutrient‐rich by‐products from the agri‐food industry offer promising avenues for disease prevention while contributing to zero‐waste goals and circular food systems.

To investigate these effects, we employed an azoxymethane (AOM) and dextran sulfate sodium (DSS) model to induce CRC in rats. AOM is metabolized in the liver to form methylazoxymethanol, a genotoxic compound that produces DNA adducts and mutations, thereby generating ROS and initiating oxidative stress. DSS, meanwhile, compromises the integrity of the colonic epithelial barrier, increases intestinal permeability, and promotes bacterial translocation, all of which trigger immune responses and elevate levels of free radicals and proinflammatory cytokines. Together, the AOM/DSS‐induced CRC model recapitulates both mutagenic and chronic inflammatory processes, particularly relevant to colitis‐associated CRC in patients with inflammatory bowel disease (IBD) (Ahmed et al. 2024; Dzhalilova et al. 2023).

Therefore, this study was designed to assess the bioaccessibility and antioxidant potential of DRB in both in vitro and in vivo CRC models while concurrently evaluating its modulatory effects on gut microbiota and redox biomarkers. By exploring DRB's role in intestinal oxidative stress and microbiome dynamics, we seek to generate mechanistic insights that support its application as a multifunctional, health‐promoting food ingredient. In addition, we expect to contribute to the revalorization of DRB within a sustainable, zero‐waste food system framework.

## 2 Materials and Methods

### 2.1 Chemicals and Materials

All chemicals and reagents utilized in the study were of analytical grade. Most of the chemicals were purchased from Sigma‐Aldrich Chemical Company, including quercetin standard, gallic acid standard, Trolox, 2,4,6‐tripyridyl‐s‐triazine (TPTZ) solution, 2,2‐diphenyl‐1‐picrylhydrazyl (DPPH), 2,2′‐azobis‐(2‐amidinopropane) dihydrochloride (AAPH), ortho‐phthalaldehyde (OPA), α‐amylase from porcine pancreas, pepsin (P7000), pancreatin (P1750), porcine bile extract (B8631), 2′,7′‐dichlorofluorescein diacetate (DCFH_2_‐DA), high‐glucose Dulbecco's Modified Eagle Medium (DMEM), 3‐(4,5‐dimethyl‐2‐thiazolyl)‐2,5‐diphenyltetrazolium bromide (MTT), and dimethyl sulphoxide (DMSO). Disodium hydrogen phosphate anhydrous (Na_2_HPO₄) was procured from RCI Labscan Company, Thailand. The heat‐inactivated fetal bovine serum (FBS), fungizone (antifungal), non‐essential amino acids, and penicillin–streptomycin (antibiotic) were obtained from GIBCO Invitrogen, Auckland, New Zealand. Aluminum chloride, disodium hydrogen phosphate, sodium lauryl sulfate, and copper sulfate hydrate were sourced from KEMAUS Company, Australia. The glutathione peroxidase assay kit was sourced from Northwest Life Science Specialities Company, USA. The Thermo Scientific Pierce BCA Protein Assay kit was purchased from Thermo Fisher Scientific Inc., USA. The Caco‐2 colon adenocarcinoma cell was acquired from the American Type Culture Collection (ATCC, Rockville, MD). Azoxymethane (AOM, cat no A5486) was purchased from Sigma‐Aldrich, Singapore. Dextran sulfate sodium (DSS, cat no 9011‐18‐1) was purchased from TdB Consultancy, Sweden.

### 2.2 Preparation of Defatted Rice Bran Powder

Defatted rice bran (DRB), a by‐product of rice bran oil production, was acquired from Thai Ruam Jai Vegetable Oil Co. Ltd. in Thailand. The DRB originated from a blend of Thai rice bran varieties ( *Oryza sativa* ) from a local rice mill in central Thailand. Rice bran oil was extracted using a solvent extraction method. To meet food‐grade standards, the DRB underwent processing, dehydration to achieve a moisture content below 6%, pulverization, sieving through a 60‐mesh sieve, and subsequent storage in airtight containers at −20°C. Prior to its application in the animal study for dietary administration, the DRB verified the safety of the material by conducting a heavy metal contamination test at an ISO/IEC 17025‐accredited laboratory. Inductively Coupled Plasma Mass Spectrometry (ICP‐MS) analysis revealed that the arsenic content in the DRB was less than 0.1 mg/kg, well below the regulatory limits established by the Codex Alimentarius (0.2 mg/kg for polished rice) and the European Commission (0.25 mg/kg for rice bran intended for human consumption). DRB was delivered to the laboratory at the commencement of the experiment.

### 2.3 Analysis of Dietary Fiber Fractions

The total dietary fiber (TDF), soluble fiber, and insoluble fiber content in defatted rice bran (DRB) were quantified using the Megazyme Total Dietary Fiber Assay Kit (K‐TDFR), following AOAC Method 991.43, AOAC 985.29, AACC 32‐07.01, and AACC 32‐05.01. Approximately 1 g of the DRB sample underwent enzymatic digestion utilizing heat‐stable α‐amylase, protease, and amyloglucosidase to simulate human digestive processes. The soluble fiber was precipitated with 95% ethanol, while the insoluble fiber was isolated through filtration, followed by washing and drying at 105°C. Both fiber fractions were subjected to ashing at 525°C to determine mineral content. The calculations for each fiber type were performed as follows: insoluble fiber = [(weight of dried residue − weight of ash)/weight of sample] × 100; soluble fiber = [(weight of soluble residue − weight of ash)/weight of sample] × 100; and TDF = insoluble fiber + soluble fiber. All measurements were conducted in triplicate to ensure precision and reliability.

### 2.4 Analysis of Antioxidant Potential in Defatted Rice Bran

#### 2.4.1 Extraction Methods

Approximately 1000 mg of the powdered sample was extracted with 10 mL of 70% methanol in water at room temperature using a vortex mixer for 24 h. Subsequently, the sample was centrifuged at 9084 *g* at 4°C for 15 min and filtered through a nylon membrane. The resulting extract was then preserved at −80°C for further analysis. All experiments were performed in triplicate.

#### 2.4.2 Determination of Total Flavonoid Content

The total flavonoid content was quantified using the aluminum chloride colorimetric assay described by Baba and Malik (2015) with slight modifications. A 1.5 μL sample (100 mg/mL) of quercetin standard was dispensed into a 96‐well plate. Subsequently, a solution comprising 30 μL MeOH, 120 μL deionized water, and 9 μL of 5% NaNO_2_ was added and incubated in darkness for 5 min. Following this, 9 μL of 10% AlCl₃ was introduced and allowed to react at room temperature without light exposure for 6 min. The reaction mixture was then neutralized with 60 μL of 1 M NaOH and 300 μL of deionized water, followed by further incubation in darkness at room temperature for 15 min. Upon completion of the reaction, the absorbance was recorded at 510 nm using a spectrophotometer. The flavonoid content was quantified as mg of quercetin equivalent (QE) per 100 g of dry matter (DM).

#### 2.4.3 Determination of Total Phenolic Contents

Total phenolic content (TPC) was determined following the method outlined by previous studies (Baba and Malik 2015; Mazzucotelli et al. 2017). TPC values were quantified in mg of gallic acid equivalents (GAE) per 100 g DM. A calibration curve was constructed using a standard gallic acid solution. In a 96‐well plate, 10 μL of a sample (100 mg/mL) was combined with 150 μL of deionized water and 25 μL of Folin–Ciocalteu reagent. After a 3‐min incubation at room temperature, 100 μL of sodium carbonate solution (75 g/L) was added, followed by a 60‐min incubation period. The absorbance was measured at 650 nm using a UV–Vis spectrophotometer. Each sample was analyzed in triplicate.

#### 2.4.4 Determination of Phytic Acid

Phytic acid levels in DRB were quantified using the Megazyme Phytic Acid (Total Phosphorus) Assay Kit (K‐PHYT) per AOAC Method 986.11 and the manufacturer's instructions. Approximately 0.5 g of finely ground rice was subjected to extraction with 0.66 M hydrochloric acid for three hours, followed by centrifugation at 12,000 *g* for ten min. The resulting supernatant underwent enzymatic hydrolysis with phytase and alkaline phosphatase at 37°C for sixty min to liberate inorganic phosphate, which was subsequently quantified spectrophotometrically at a wavelength of 655 nm. The phytic acid content was determined based on the amount of phosphate released, employing a standard curve and a molecular weight conversion factor. All analyses were conducted in triplicate.

#### 2.4.5 Ferric Reducing Antioxidant Power (FRAP) Assay

The FRAP assay was performed according to the protocol outlined by Chaikham and Prangthip (2015). Samples and standards were analyzed in triplicate. To prepare the FRAP reagent, a mixture of 300 mM sodium acetate buffer (pH 3.6, 10 mL), 10 mM 2,4,6‐tripyridyl‐S‐triazine (TPTZ) solution in 40 mM hydrochloric acid (1 mL), and 20 mM FeCl_3_·6H_2_O (1 mL) was used. Subsequently, the sample (20 μL) was mixed with the FRAP reagent (150 μL) in a 96‐well plate to initiate the reaction at 37°C. Following a 30 min incubation period, the absorbance at 593 nm was determined using a microplate reader. The FRAP values, denoted in mg Trolox per 100 mg, were computed based on the standard curve.

#### 2.4.6 2,2‐Diphenyl‐1‐Picrylhydrazyl (DPPH) Radical‐Scavenging Activity Assay

The assessment of antioxidant activity was conducted through the DPPH radical‐scavenging assay, as modified from the previous methods (Hale et al. 2008; Liao et al. 2012). A 50 μL aliquot of the sample solution was combined with 100 μL DPPH solution in a 96‐well plate. The mixture was then incubated for 30 min in darkness at room temperature, followed by the absorbance measurement at 517 nm using a microplate reader. Subsequently, the DPPH values in mg Trolox per 100 mg were determined based on the standard curve.

#### 2.4.7 Oxygen Radical Antioxidant Capacity (ORAC) Assay

The experiment was carried out following a prior investigation (Oueslati et al. 2011) with minor adjustments. Fifteen μL of the sample (1 μg/mL) was dispensed into a black 96‐well plate, followed by 75 μL of fluorescein solution (70 nM final concentration) in a phosphate buffer (pH 7.4, 75 mM). Peroxyl radicals were generated by adding 15 μL of 40 mM AAPH. Subsequently, the plate was positioned in a spectrophotometer with fluorescence detection set at excitation and emission wavelengths of 485 and 540 nm, respectively. Fluorescence measurements were recorded every 2 min over 60 min at 37°C. Trolox (0–50 μM) was utilized as a standard inhibitor. The sample's area under the curve (AUC) was calculated, and the outcomes were presented as μM Trolox equivalent (TE) per 100 mg.

### 2.5 Determination of Bioaccessibility of Total Flavonoid Content (TFC) and Total Phenolic Content (TPC) in Defatted Rice Bran

The simulated gastrointestinal digestion was carried out using modified protocols that included porcine‐derived enzymes, specifically pepsin, pancreatin, and bile extract, to mimic human digestion, following established methodologies widely accepted for in vitro bioaccessibility studies (Brodkorb et al. 2019; Minekus et al. 2014). Simulated salivary fluid (SSF), gastric fluid (SGF), and intestinal fluid (SIF) were freshly prepared according to the standard compositions outlined in the protocol. All digestion steps were performed at 37°C with gentle agitation.

For the oral phase, 2 g of defatted rice bran (DRB) powder was mixed with 1 mL of deionized water, 2.1 mL of SSF, 0.3 mL of α‐amylase solution (75 U/mL), 155 μL of CaCl_2_ (0.3 M), and 585 μL of deionized water. The pH was adjusted and maintained at 7.0 using 1 M NaOH before incubation at 37°C for 2 min to simulate mastication.

In the gastric phase, 6 mL of the oral digest was mixed with 4 mL of SGF, 960 μL of pepsin solution (25,000 U/mL), and 3 μL of CaCl_2_ (0.3 M). The pH was adjusted to 3.0 using 1 M HCl, and the total volume was adjusted to 10 mL with deionized water. The mixture was then incubated at 37°C for 2 h to simulate gastric digestion.

For the intestinal phase, 12 mL of the gastric chyme was mixed with 6.6 mL of SIF, 3 mL of pancreatin (800 U/mL trypsin activity), 1.5 mL of bile extract (0.38 g/mL in SIF), 24 μL of CaCl_2_ (0.3 M), and 90 μL of NaHCO₃ (1 M). The pH was adjusted to 7.0 using 1 M NaOH, and the total volume was brought to 20 mL with deionized water. The mixture was incubated at 37°C for 2 h to complete intestinal digestion.

After digestion, the samples were immediately cooled on ice to halt enzymatic activity and then centrifuged at 10,000 *g* for 10 min at 10°C. The resulting supernatants were filtered through 0.45 μm membranes and stored at −20°C until further analysis. A blank control and an enzyme‐free control were included in all digestion steps. All analyses were performed in triplicate.

The bioaccessible fractions obtained after the intestinal phase were analyzed for total flavonoid content (TFC) and total phenolic content (TPC) as described in Sections 2.4.2 and 2.4.3. Bioaccessibility was calculated using the following formula: Bioaccessibility = (Concentration in bioaccessible fraction/Concentration in undigested DRB) × 100.

Results are expressed as the percentage of TFC and TPC released into the soluble fraction during digestion, relative to the initial content in DRB.

### 2.6 The Study Investigated the Effects of Digested Defatted Rice Bran on Oxidative Stress Conditions in Caco‐2 Cells

#### 2.6.1 Cell Culture

Caco‐2 cells (passages 10–30) were cultured in Dulbecco's Modified Eagle Medium (DMEM), supplemented with 1% penicillin–streptomycin, 1% L‐glutamine, 1% non‐essential amino acids, 0.2% fungizone, and 15% fetal bovine serum (FBS), at 37°C with 5% CO_2_. Upon reaching confluence, cells were maintained in a medium containing 7.5% FBS. Cells were seeded at 1 × 10^4^ cells/well in black 96‐well plates (100 μL medium/well) and incubated for 24 h. Cell viability and ROS assays were conducted after microscopic cell adherence and confluence verification.

#### 2.6.2 MTT Cell Viability Assay

Preliminary MTT (3‐(4,5‐Dimethyl‐2‐Thiazolyl)‐2,5‐Diphenyltetrazolium Bromide) assays (José Ruiz et al. 2006) were performed using dilution ratios of 1:3, 1:4, and 1:5. The 1:3 dilution was the highest concentration that did not exhibit cytotoxicity in Caco‐2 cells (*p* ≥ 0.05 compared to control) and was thus selected for subsequent assays. Although this study focused on non‐toxic conditions, further research is warranted to explore dose‐dependent effects.

#### 2.6.3 Intracellular Antioxidant (DCFH_2_ ‐DA) Assay

Intracellular antioxidant activity of digested DRB was assessed using the fluorescent probe 2′,7′‐dichlorofluorescein diacetate (DCFH_2_‐DA), adapted from Alía et al. (2005). Caco‐2 cells (5 × 10^5^ cells/well) were cultured overnight in 6‐well plates at 37°C in a 5% CO_2_ incubator. Cells were treated with digested DRB (0.2 mL/well, non‐toxic concentration) in complete DMEM for 4 h. Subsequently, cells were exposed to either culture medium (positive control) or 0.5 mM hydrogen peroxide (H_2_O_2_; negative control) for 30 min, followed by incubation with 10 ng/mL IL‐1β for 20 h. Cells were washed with phosphate‐buffered saline (PBS, pH 7.4), incubated in the dark with 100 μL of 10 μM DCFH_2_‐DA for 30 min, rinsed, and resuspended in PBS. Fluorescence intensity (excitation: 485 nm; emission: 535 nm) was measured to quantify cellular ROS levels.

#### 2.6.4 Intracellular Anti‐Inflammatory Assay

The anti‐inflammatory activity of digested DRB was evaluated by measuring cytokine secretion (IL‐6, IL‐8, TNF‐α) in Caco‐2 cells. Cells were treated with digested DRB (0.2 mL/well, non‐toxic concentration) or culture medium (positive control) for 4 h. Cells were then washed and exposed to 0.5 mM hydrogen peroxide (H_2_O_2_) for 30 min, followed by incubation with 10 ng/mL interleukin‐1 beta (IL‐1β) for 20 h at 37°C to induce oxidative stress. Cytokine concentrations in the culture media were determined using enzyme‐linked immunosorbent assay (ELISA). The percentage inhibition of cytokine secretion was calculated as % inhibition = 100 × [(Control Cytokine Secretion − Sample Cytokine Secretion)/Control Cytokine Secretion].

### 2.7 The Study Investigated the Effect of Low and High Doses of DRB in AOM/DSS‐Induced Oxidative Stress in Rats

#### 2.7.1 Animals and Experimental Design

This study provides insights into the potential of DRB to mitigate oxidative stress in the colon, modeled through azoxymethane (AOM) and dextran sodium sulfate (DSS) induction, reflecting its relevance to human dietary studies concerning oxidative stress and colon health. The research investigated the antioxidative effects of DRB on oxidative stress in rat colons induced by AOM/DSS. This experiment was conducted following the ethical and scientific care guidelines approved by the Siriraj Animal Care and Use Committee at Mahidol University (COA no. 004/2562). Male Wistar rats, procured from Nomura Siam International Co. Ltd., Bangkok, were maintained in controlled environmental conditions (23°C ± 1°C and a 12‐h light/dark cycle), with free access to food and water.

**FIGURE 1:**
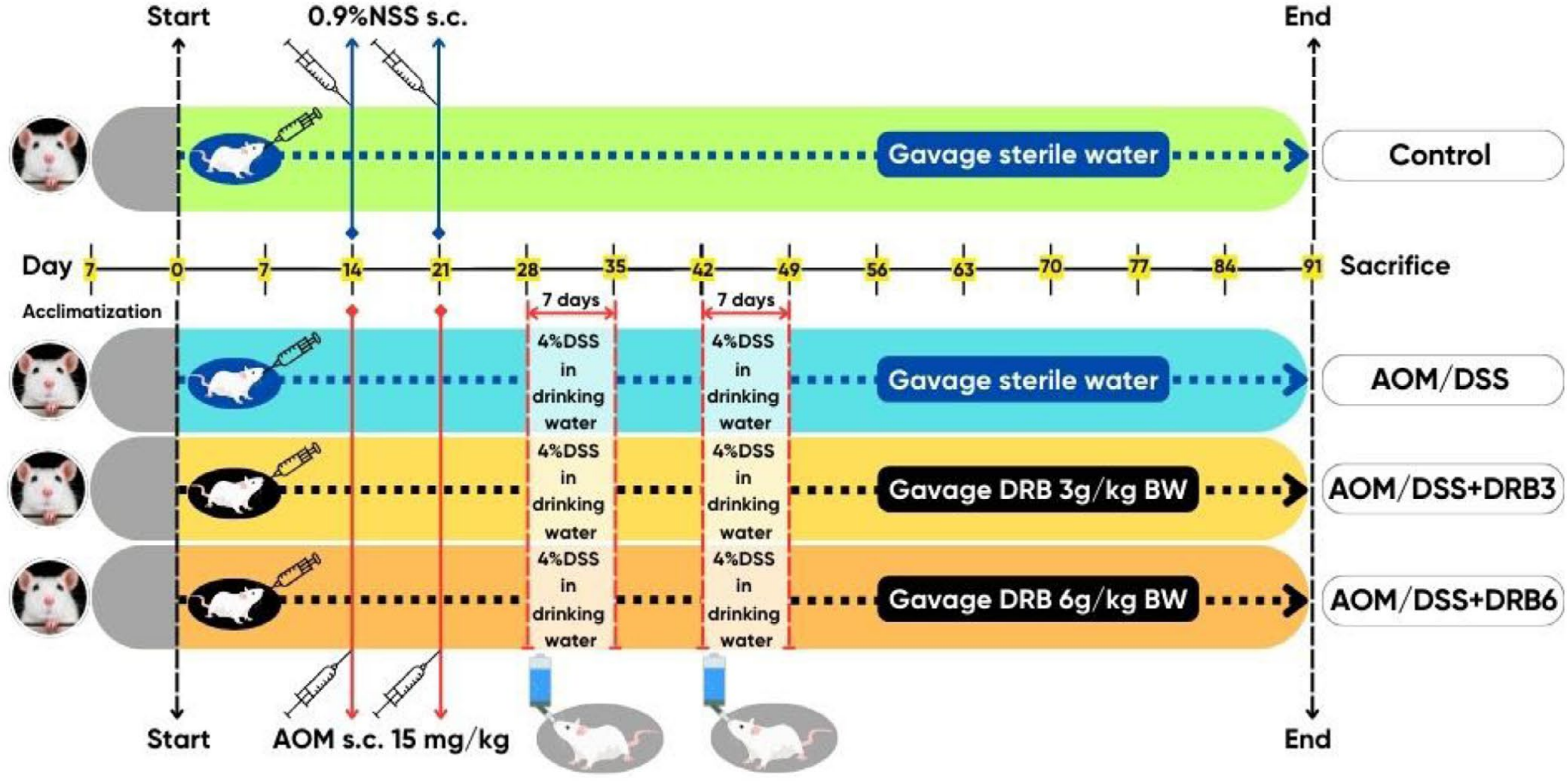
The illustration presents the study design employed to evaluate the antioxidant effects of low‐dose and high‐dose defatted rice bran (DRB) in an experimental rat model of AOM/DSS‐induced colon oxidative stress. After a seven‐day acclimation period, the rats in the control and azoxymethane/dextran sodium sulfate (AOM/DSS) groups were administered sterile water via gavage, while the rats in the AOM/DSS + DRB3 and AOM/DSS + DRB6 groups received DRB powder at doses of 3 and 6 g/kg body weight (BW), respectively, dissolved in sterile water. This treatment was administered once daily throughout the experimental period, from day 1 to day 90. The rats in the AOM/DSS, AOM/DSS + DRB3, and AOM/DSS + DRB6 groups were subcutaneously injected (s.c.) with AOM at a dosage of 15 mg/kg BW on days 14 and 21. Subsequently, these groups were given 4% DSS in their drinking water on two separate occasions, from days 26 to 35 and 42 to 49 (for seven days each time). In contrast, the rats in the control group received subcutaneous injections of 0.9% normal saline solution (NSS) on days 14 and 21.

Following a one‐week acclimatization period, animals were randomly divided into four groups (*n* = 5 per group). Group 1 (control) and Group 2 (AOM/DSS) received daily oral gavage of sterile water (1 mL). Groups 3 (AOM/DSS + DRB3) and 4 (AOM/DSS + DRB6) were administered DRB at 3 and 6 g/kg body weight, respectively, by oral gavage for the entire study duration (Figure 1). These doses were selected based on previous studies reporting daily intake of 30 g of DRB in healthy individuals and colorectal cancer survivors (Brown et al. 2017; Sheflin et al. 2015). The human dosage (mg/kg BW) was converted to an equivalent animal dosage using body surface area (BSA) ratios, with reference weights of 60 kg for humans and 200 g for rats (Nair and Jacob 2016). To further support translational relevance, the 6 g/kg dose in rats was converted to a human equivalent dose (HED) using the BSA normalization method (U.S. Food and Drug Administration 2005). The correction factor (K_m_) for rats is 6, and for adults, it is 37. This results in an HED of approximately 0.97 g/kg, or around 58 g/day for a 60‐kg human, which remains within the range of dietary intake feasible for nutritional interventions.

The sample size was determined based on protein carbonyl levels reported by Cid‐Gallegos et al. (2020) in a comparable AOM/DSS rat model. The pooled standard deviation was 2.1, resulting in a Cohen's d of 1.48. Power analysis using G*Power 3.1 (two‐tailed *t*‐test, *α* = 0.05, power = 0.80) showed that five rats per group are sufficient. This group size complies with ethical guidelines based on the 3Rs principle (see Table S1).

On days 14 and 21, AOM was administered subcutaneously at a dose of 15 mg/kg to all groups, except the control group, which received 0.9% normal saline. From days 26 to 35 and 42 to 49, all non‐control groups received 4% DSS (w/v) in drinking water for two separate 7‐day periods. After a 90‐day experimental period, rats were euthanized by CO_2_ asphyxiation. Blood was collected by cardiac puncture, centrifuged at 1500 *g* for 15 min at 4°C, and serum was stored at −80°C. Colonic tissues were snap‐frozen in liquid nitrogen and stored at −20°C for further analysis.

#### 2.7.2 Sample Collection and Colon Homogenate Preparation

Colon tissues (1 g) were homogenized in phosphate‐buffered saline (PBS, pH 7.4; 10 mL) using a homogenizer. The homogenates were centrifuged at 13,000 *g* for 10 min at 4°C, and supernatants were collected and stored at −80°C until analysis. Oxidative stress markers, including reduced glutathione (GSH) content and antioxidant enzyme activities (glutathione peroxidase [GPx], superoxide dismutase [SOD], and catalase [CAT]), were assessed. Protein concentrations were determined by the bicinchoninic acid (BCA) assay, using bovine serum albumin as the standard (Smith et al. 1985).

#### 2.7.3 Determination of Reduced Glutathione

Reduced glutathione (GSH) concentrations in colon tissue homogenates were measured using fluorescence spectroscopy, adapted from previously described methods (Hissin and Hilf 1976; Wunjuntuk et al. 2016) with minor modifications. Briefly, tissues were homogenized in ice‐cold phosphate‐buffered saline (PBS, pH 7.4), centrifuged at 10,000 *g* (15 min, 4°C), and the supernatant was collected. Aliquots were mixed with o‐phthalaldehyde (OPA) reagent (100 mM phosphate buffer, pH 8.0) and incubated in the dark at room temperature for 15 min. Fluorescence intensity was measured (excitation: 350 nm, emission: 420 nm). GSH concentrations were calculated from a standard curve and expressed as μmol/g tissue. Analyses were performed in triplicate.

#### 2.7.4 Determination of Antioxidant Enzyme Activities

Activities of glutathione peroxidase (GPx), superoxide dismutase (SOD), and catalase (CAT) were assessed using commercial assay kits according to manufacturers' guidelines. GPx activity (Northwest Life Science, Vancouver, WA, USA) was determined by measuring NADPH oxidation at 340 nm. SOD activity (Cayman Chemical, Ann Arbour, MI, USA) was quantified by measuring the inhibition of tetrazolium salt reduction at 450 nm. CAT activity (Cayman Chemical, Ann Arbour, MI, USA) was measured by spectrophotometric formaldehyde detection at 540 nm using Purpald reagent. Enzyme activities were normalized to protein content and expressed as units per mg of protein (U/mg protein). All assays were performed in triplicate.

#### 2.7.5 Determination of Protein Carbonyl Level

Protein carbonyl levels, indicators of oxidative protein damage, were measured using an enzyme‐linked immunosorbent assay (ELISA) kit (Abcam, Cambridge, UK). Colon tissue homogenates were prepared in phosphate‐buffered saline (PBS, pH 7.4), centrifuged (10,000 *g*, 15 min, 4°C), and supernatants collected. Samples were derivatized with 2,4‐dinitrophenylhydrazine (DNPH) and incubated in ELISA plates pre‐coated with anti‐dinitrophenyl (DNP) antibodies. Detection was achieved with horseradish peroxidase (HRP)‐conjugated secondary antibodies, and absorbance was read at 450 nm. Carbonyl content was quantified using a standard curve, normalized to protein concentrations determined by the bicinchoninic acid (BCA) assay, and expressed as nmol/mg protein. All assays were performed in triplicate.

#### 2.7.6 Determination of 8‐Hydroxy‐2‐Deoxyguanosine (8‐OHdG)

Total DNA was extracted from colon tissue utilizing a DNA extraction kit (Qiagen, Hilden, Germany). The level of 8‐hydroxy‐2‐deoxyguanosine (8‐OHdG) in the colon was quantified using an ELISA kit (Abcam, UK). The data were expressed in nanograms per mg of DNA. The concentration of DNA was determined using a Thermo Fisher Scientific NanoDrop 2000c Spectrophotometer (Waltham, MA, USA).

### 2.8 Gut Microbiota Data Analysis

#### 2.8.1 Sample Collection and DNA Extraction

Colonic luminal (fecal) and mucosal samples were collected from all rat groups under sterile conditions and were immediately stored at −20°C to maintain microbial integrity. Metagenomic DNA was extracted utilizing the QIAamp PowerFecal Pro DNA Kit (Qiagen, Germantown, MD, USA), adhering to the manufacturer's protocol with modifications implemented to enhance microbial cell lysis. A bead‐beating step was conducted at 5000 x g for one minute and repeated twice with cooling intervals before the chemical lysis using proprietary buffers. Contaminants were eliminated through successive wash steps, and the DNA was subsequently eluted in a low‐EDTA Tris buffer and stored at −20°C. Negative controls (reagent‐only) were incorporated to monitor potential contamination.

#### 2.8.2 DNA Purification and Quality Assessment

The concentration and purity of DNA were evaluated using a NanoDrop spectrophotometer (Implen GmbH, München, Germany), employing the absorbance ratios of A260/A280 and A260/A230. The integrity of the DNA was confirmed through 1% agarose gel electrophoresis, which ensured the presence of high molecular weight and minimal degradation. The purified DNA was aliquoted and stored at −20°C for future applications.

#### 2.8.3 16S rRNA Gene Amplification and Library Preparation

The V3–V4 regions of the bacterial 16S rRNA gene were amplified using primers 341F and 805R. PCR reactions (25 μL) contained DNA template (5–10 ng), primers (10 μM each), nuclease‐free water, and 2× sparQ HiFi PCR Master Mix (Quantabio, USA). Thermocycling conditions included initial denaturation (98°C, 2 min), 28 cycles of denaturation (98°C, 20 s), annealing (60°C, 30 s), extension (72°C, 1 min), and a final extension (72°C, 1 min). PCR products (~460 bp) were verified by agarose gel electrophoresis and purified using sparQ PureMag beads. Indexed libraries were generated using the Nextera XT Index Kit v2 (Illumina, USA) through an additional 8‐cycle PCR. Libraries were purified, quantified (Qubit dsDNA HS Assay Kit, Invitrogen), normalized to 4 nM, pooled, diluted to 5 pM, and sequenced with 25% PhiX spike‐in on an Illumina MiSeq platform (2 × 250 bp; MiSeq Reagent Kit v2) at the Omics Sciences and Bioinformatics Centre, Chulalongkorn University, Bangkok, Thailand.

#### 2.8.4 Sequence Processing and Taxonomic Classification

Raw sequencing reads were processed using the QIIME2 pipeline, which included quality filtering, trimming, and chimaera removal through the DADA2 algorithm. Operational Taxonomic Units (OTUs) were clustered at a similarity threshold of 97%, and taxonomic classification was performed using the SILVA database (version 138). The abundances of a specific microorganism within the microbial community were subsequently calculated for further analyses. Data visualization, including the creation of circular plots, was conducted using GraphPad Prism.

#### 2.8.5 Microbial Network Analysis and Correlation Patterns

Microbial network analysis explored interactions among genera within fecal and mucosal microbiota. Co‐occurrence networks were constructed based on relative abundances at the genus level, with significant pairwise correlations (*p* < 0.05, |*r*| > 0.6) computed using Spearman's correlation and visualized in Cytoscape (version 3.8.2). Key microbial taxa were identified using network‐centrality metrics, including degree, closeness, and betweenness centrality, via the NetworkAnalyzer plugin in Cytoscape. Genera with a high degree of centrality and closeness centrality were classified as potential keystone taxa. Comparative analyses assessed network topology changes across experimental groups in response to dietary interventions. Heatmaps of correlation patterns were generated in R (version 4.1.2) using the pheatmap package. Significant correlations (*p* < 0.05) were color‐coded, with green for positive and red for negative correlations, providing insights into microbial interactions within fecal and mucosal communities.

### 2.9 Quantification of Short‐Chain Fatty Acids (SCFAs)

Short‐chain fatty acids (SCFAs), specifically acetic, propionic, and butyric acids, were quantified in cecal samples using gas chromatography with flame‐ionization detection (GC‐FID), following the methodology of Ribeiro et al. (2018) and subsequently standardized according to the protocol of Tajasuwan et al. (2023). The method was rigorously validated in accordance with the International Council for Harmonization (ICH) requirements for bioanalytical method validation.

Approximately 20 mg of cecal material was aseptically transferred into microcentrifuge tubes and immediately kept at −20°C for future analysis. To extract SCFAs, each sample was homogenized in 200 μL of distilled water and mixed with 20 mg citric acid, 40 mg sodium chloride (NaCl), 40 μL of 0.1 M hydrochloric acid (HCl), and 200 μL of an organic solvent mixture comprising n‐butanol, tetrahydrofuran, and acetonitrile in a 5:3:2 volume ratio. Isocaproic acid was added at a final concentration of 0.5 mM as a reference substance because it behaves similarly to the target SCFAs during the testing process. The mixture was vortexed for 1 min, incubated on ice for 30 min, and subsequently centrifuged at 15,000 *g* for 10 min at 4°C. The supernatant was filtered using a 0.22 μm polytetrafluoroethylene (PTFE) syringe filter and then placed into autosampler vials.

SCFA analysis was conducted using an Agilent 6890 N gas chromatograph equipped with a flame‐ionization detector and a DB‐23 capillary column (60 m, 0.25 mm i.d., 0.25 μm film thickness, including 74.5% 1‐methylnaphthalene). Helium functioned as the carrier gas at a constant flow rate of 1.2 mL/min, with a split injection ratio of 50:1 and an injection volume of 2 μL. The oven temperature schedule was specified as follows: commence with a 5‐min hold at 70°C, then increase to 80°C at a rate of 5°C/min (maintained for 5 min), followed by a rise to 200°C at 5°C/min (kept for 2 min). The detector temperature was maintained at 250°C. Quantification was performed using external standard calibration curves (0.1–10 mM, Sigma‐Aldrich), with peak regions normalized to the internal standard. All measurements were conducted in triplicate to ensure analytical accuracy. The approach exhibited validation for linearity (*R* ^2^ > 0.99 over the calibration range) and precision (coefficient of variation < 5%). Recovery rates were established by augmenting cecal samples with known quantities of SCFA standards, resulting in recoveries ranging from 90% to 110%. The limits of detection (LOD) ranged from 0.05 to 0.10 mM, whereas the limits of quantitation (LOQ) varied from 0.10 to 0.25 mM for the three short‐chain fatty acids (SCFAs). The values were determined using routine addition in fecal matrices and confirmed according to ICH Q2(R1) criteria, affirming the method's sensitivity and robustness. SCFA concentrations were measured in mmol/kg of cecal material.

Group‐specific SCFA patterns were illustrated by depicting the relative proportions in a mirrored bar chart. Principal component analysis (PCA) was conducted using standardized SCFA concentrations to assess multivariate disparities among experimental groups. A covariance matrix was used for PCA, and the first two principal components (PC1 and PC2), with eigenvalues greater than one, were examined to identify clustering patterns and metabolic differentiation related to microbial activity.

### 2.10 Correlation Analysis

Spearman's rank correlation (Spearman 1904) was employed to assess associations among gut microbiota composition, short‐chain fatty acids (acetic, butyric, and propionic acids), and redox‐related biomarkers, including antioxidant enzymes (GSH, SOD, and CAT) and oxidative stress markers (8‐OHdG and protein carbonyl). All variables were log‐transformed to reduce skewness and stabilize variance. Correlation strength was classified as weak (|*r*| < 0.4), moderate (|*r*| = 0.4–0.6), or strong (|*r*| > 0.6), based on Cohen's benchmarks (Cohen 2013). Multiple comparisons were corrected using the Benjamini–Hochberg false discovery rate (FDR) method (Benjamini and Hochberg 1995). Statistically significant associations (FDR‐adjusted *p* < 0.05) were visualized using heatmaps and correlation network diagrams.

### 2.11 Statistical Analysis

All statistical analyses were performed using GraphPad Prism (version 10.4.0; GraphPad Software, San Diego, CA, USA). Data from in vitro experiments and cell culture assays are expressed as mean ± standard deviation (SD). Between‐group comparisons were evaluated using the Student's *t*‐test for normally distributed data and the Mann–Whitney *U* test for non‐normally distributed data. For in vivo animal experiments, results are presented as mean ± standard error of the mean (SEM). Statistical differences among multiple groups were analyzed using one‐way analysis of variance (ANOVA) followed by Tukey's post hoc test for multiple comparisons. A *p* value < 0.05 was considered statistically significant.

## 3 Results

### 3.1 Total Dietary Fiber, Insoluble Dietary Fiber, and Soluble Dietary Fiber

As presented in Table 1, this study's findings indicate that DRB contains 28.3 ± 0.3 g of total dietary fiber (TDF), 21.8 ± 0.8 g of insoluble dietary fiber (IDF), and 6.5 ± 0.4 g of soluble dietary fiber (SDF) per 100 g of dry matter (DM). DRB exhibits a moisture content of 6.12 g per 100 g.

**TABLE 1:** Dietary fiber content, bioactive compounds and antioxidant capacities of defatted rice bran (DRB).

DRB compositions	Content
Moisture content (g/100 g)	6.12
**Bioactive compounds**	**Per 100 g DM of DRB**
Total dietary fiber, TDF (g)	28.3 ± 0.3
Insoluble dietary fiber, IDF (g)	21.8 ± 0.8
Soluble dietary fiber, SDF (g)	6.5 ± 0.4
Total flavonoid content, TFC (mg QE)	10.9 ± 0.2
Total phenolic content, TPC (mg GAE)	312.0 ± 0.4
Phytic acid (g)	8.0 ± 0.1
**Antioxidant capacities**	**Per 100 g DW of DRB**
DPPH (μmol TE)	368.6 ± 11.6
FRAP (μmol TE)	2599.1 ± 240.3
ORAC (μmol TE)	13,257.2 ± 1084.3

Abbreviations: DM, dry matter; DW, dry weight; GAE, gallic acid equivalent; QE, quercetin equivalent; TE, trolox equivalent.

### 3.2 Total Flavonoid Content (TFC), Total Phenolic Content (TPC), and Phytic Acid

The TFC, TPC, and phytic acid of DRB are shown in Table 1. DRB has a TFC content of 10.9 ± 0.2 mg QE/100 g DM, a TPC content of 312.0 ± 0.4 mg GAE/100 g DM, and a phytic acid content of 8.0 ± 0.1 g/100 g DM.

### 3.3 DPPH, FRAP, and ORAC Assays

The antioxidant activities of DRB were evaluated using three different mechanisms of action, as indicated in Table 1. For the DPPH scavenging assay, the value of DRB was 368.6 ± 11.6 μmol TE/100 g dry weight (DW). The FRAP assay's DRB value was 2599.1 ± 240.3 μmol TE/100 g DW. For the ORAC assay, the DRB value was 13,257.2 ± 1084.3 μmol TE/100 g DW.

### 3.4 Simulated Gastrointestinal Digestion and Bioaccessibility

**FIGURE 2:**
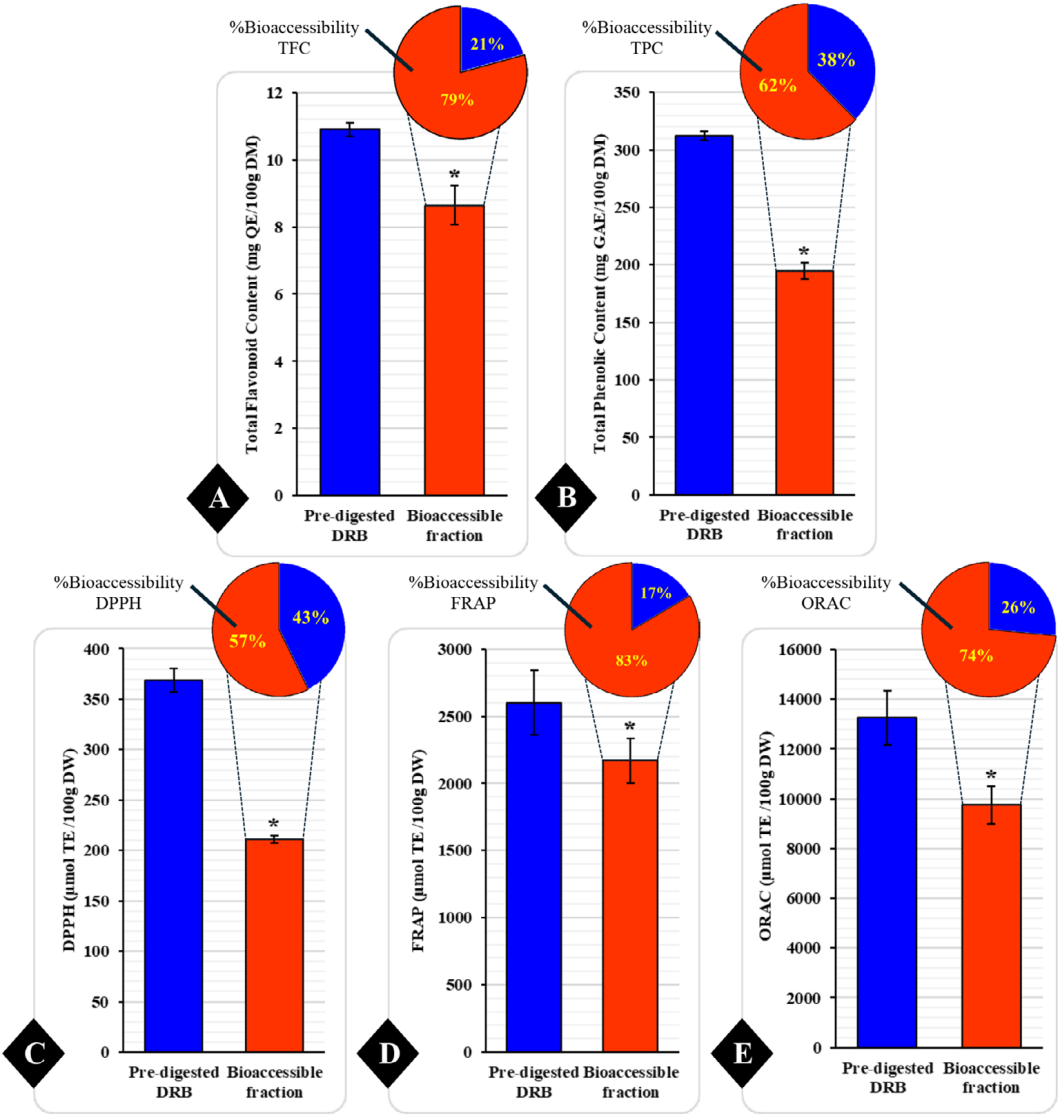
Bioaccessible fractions of total flavonoid content, total phenolic content, and antioxidant activity (DPPH, FRAP, ORAC) after simulated gastrointestinal digestion of defatted rice bran (DRB). This figure presents the total flavonoid content (A), total phenolic content (B), and antioxidant activities, including DPPH (C), FRAP (D), and ORAC (E), derived from pre‐digested defatted rice bran (DRB) (blue bar) and the bioaccessible fraction of DRB following the in vitro gastrointestinal digestion process (red bar). Data are expressed as means ± standard deviation (SD) with a sample size of *n* = 6. Statistical significance was evaluated using paired *t*‐tests, with *p < 0.05 denoting statistically significant differences.

The bioaccessibility of DRB in terms of TFC, TPC, and antioxidant activities was measured after the in vitro gastrointestinal digestion assay (Figure 2). The results showed a significant reduction of TFC (Figure 2A), TPC (Figure 2B), and antioxidant activities, including DPPH (Figure 2C), FRAP (Figure 2D), and ORAC (Figure 2E), in DRB after the simulated human gastrointestinal digestion process (*p* < 0.05). The bioaccessible fraction of DRB had a higher percentage of bioaccessibility for TFC (79%), followed by TPC (62%). Similarly, antioxidant activities indicated 74% of ORAC, followed by FRAP (83%) and DPPH (57%).

### 3.5 The Effects of Digested DRB on Antioxidant and Anti‐Inflammatory Activities in Caco‐2 Cells

#### 3.5.1 The Level of Intracellular ROS

**FIGURE 3:**
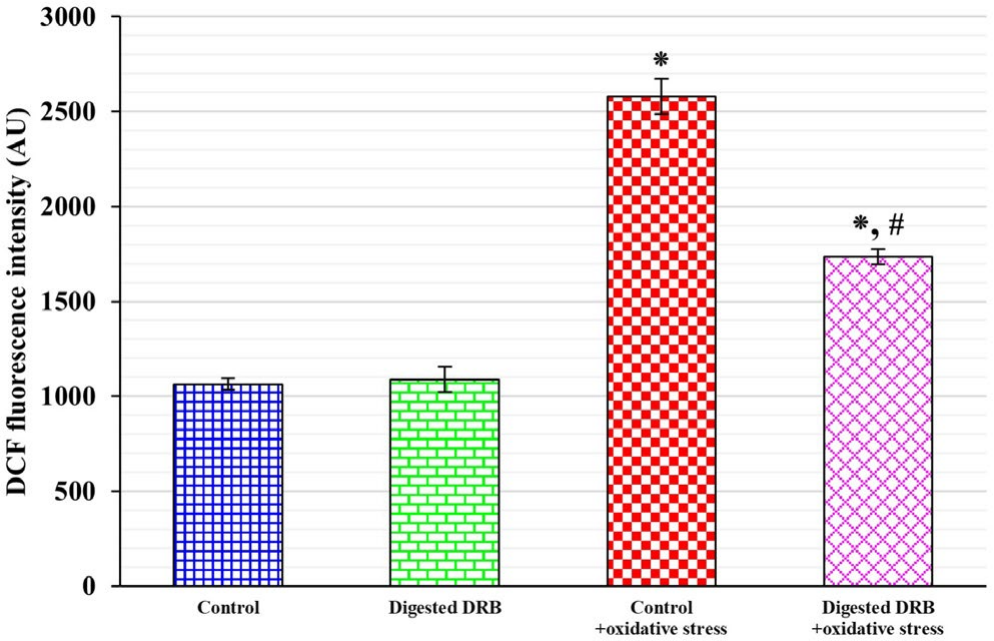
Intracellular ROS production. Caco‐2 cells were treated with the control or the digested DRB (digested defatted rice bran) for 4 h. Subsequently, the cells were incubated with or without 0.5 mM hydrogen peroxide (H_2_O_2_) for 30 min, followed by exposure to 10 ng/mL interleukin‐1 beta (IL‐1β) for 20 h to induce oxidative stress. Data are presented as mean ± standard deviation (SD) from three independent experiments conducted in triplicate (*n* = 6). Statistical significance is denoted as *p < 0.05 compared to the control and ^**#**^ *p* < 0.05 compared to the control + oxidative stress.

As illustrated in Figure 3, the intracellular ROS levels of the Caco‐2 cells exposed to H_2_O_2_ and IL‐1β demonstrated a statistically significant increase of 42.5% (*p* < 0.05) compared to the control. Conversely, when cells were pre‐treated with digested DRB before the induction of oxidative stress, the fluorescence intensity exhibited a decrease of 32.7%, a change that was also statistically significant (*p* < 0.05).

#### 3.5.2 The Level of Proinflammatory Cytokines

**FIGURE 4:**
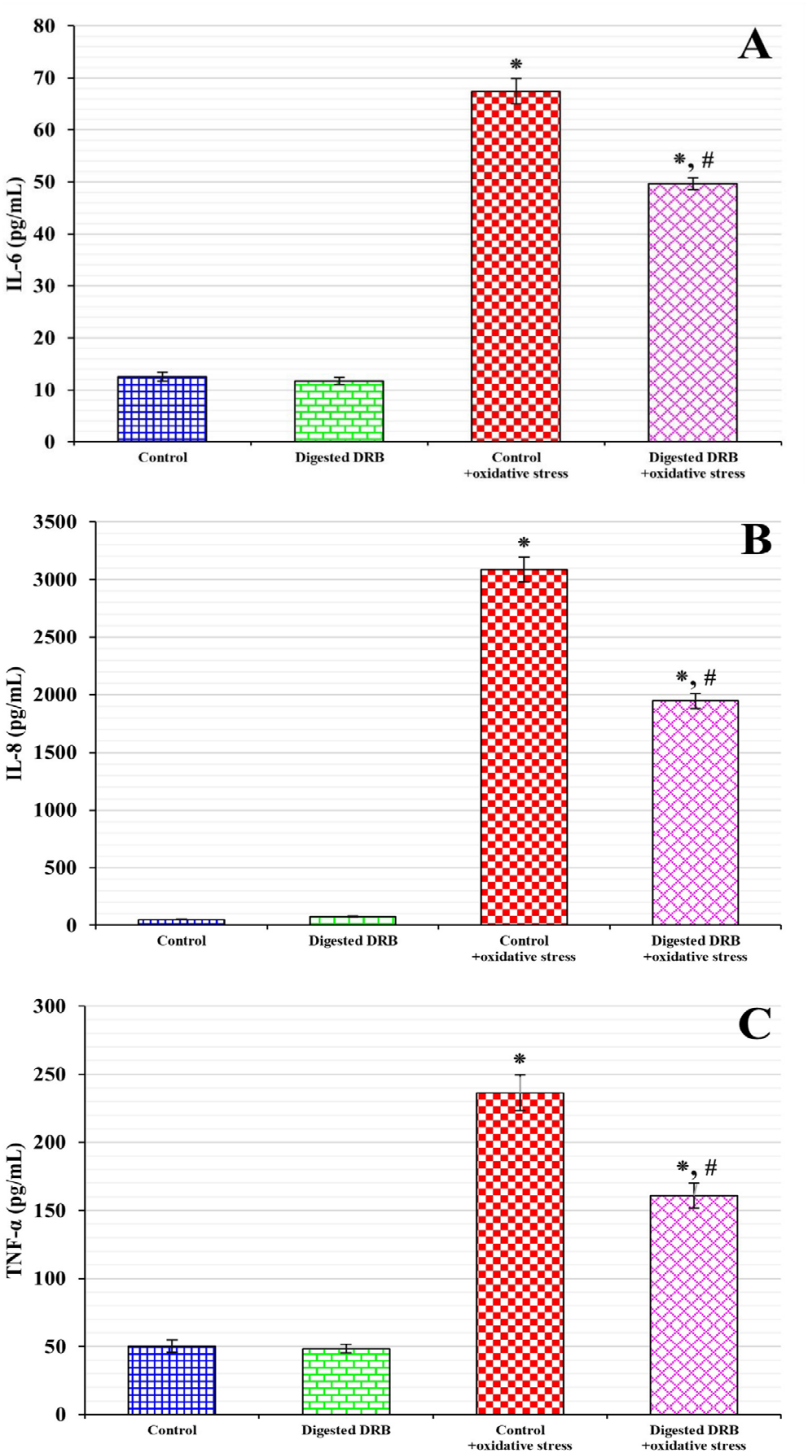
Proinflammatory cytokines, IL‐6, IL‐8, and TNF‐α. Caco‐2 cells were treated with either the control or the digested defatted rice bran (Digested DRB) for 4 h. Subsequently, the cells were incubated with or without 0.5 mM hydrogen peroxide (H_2_O_2_) for 30 min, followed by exposure to 10 ng/mL interleukin‐1 beta (IL‐1β) for 20 h to induce oxidative stress. Proinflammatory cytokine levels, including interleukin‐6 (IL‐6) (A), interleukin‐8 (IL‐8) (B), and tumor necrosis factor‐alpha (TNF‐α) (C), were measured using enzyme‐linked immunosorbent assay (ELISA). Data are presented as mean ± standard deviation (SD) from three independent experiments, each conducted in triplicate (*n* = 6). Statistical significance is indicated as *p < 0.05 compared to the control and ^**#**^ *p* < 0.05 compared to the control + oxidative stress.

As illustrated in Figure 4, there was a significant increase in proinflammatory cytokines IL‐6 (Figure 4A), IL‐8 (Figure 4B), and TNF‐α (Figure 4C) (*p* < 0.05) in Caco‐2 cells subjected to H_2_O_2_ and IL‐1β compared to the control. In contrast, the digested DRB group exhibited significantly reduced levels of IL‐6 (26.3%), IL‐8 (37.0%), and TNF‐α (32.0%) compared to the control + oxidative stress group (*p* < 0.05).

### 3.6 The Effects of DRB on AOM/DSS‐Induced Oxidative Stress in Rat Colon

To verify the metabolic safety of long‐term DRB administration, complete blood count (CBC), blood glucose, and body weight were measured in a parallel study in healthy control rats (without AOM/DSS treatment) that found DRB supplementation for 90 days did not significantly affect abnormal CBC, blood glucose levels, and other metabolic indicators, indicating no toxic and adverse glycemic effects (Tajasuwan et al. 2022). These findings support DRB's metabolic safety and usefulness as a long‐term colorectal cancer prevention diet.

#### 3.6.1 The Effects of DRB on Protein and DNA Oxidation in the Colon of Rats Subjected to AOM/DSS‐Induced Oxidative Stress

As presented in Table 2, the rats in the AOM/DSS group, AOM/DSS + DRB3 group, and AOM/DSS + DRB6 group exhibited significantly elevated levels of protein carbonyl, a biomarker indicative of protein oxidation, and 8‐OHdG, a biomarker associated with DNA oxidation, compared to the control group (*p* < 0.05). However, rats supplemented with a low dose of 3 g DRB (the AOM/DSS + DRB3 group) and a high dose of 6 g DRB (the AOM/DSS + DRB6 group) showed lower protein carbonyl and 8‐OHdG levels compared to the AOM/DSS group. Among the two different dosages of DRB, a higher dose showed significantly lowered protein and DNA oxidation (*p* < 0.05).

**TABLE 2:** The effects of defatted rice bran (DRB) on oxidative damage and antioxidant markers in the colon of rats subjected to AOM/DSS‐induced oxidative stress.

Parameters	Group			
	Control	AOM/DSS	AOM/DSS + DRB3^a^	AOM/DSS + DRB6^b^
Protein carbonyl (nmol/mg protein)	0.54 ± 0.09	13.80 ± 2.67*	9.39 ± 4.30*	4.05 ± 1.83* ^,^ ^#^
8‐OHdG (ng/mg DNA)	8.8 ± 1.2	26.3 ± 4.7*	24.0 ± 2.7*	18.6 ± 1.8* ^,^ ^#^
GSH (μg/mg protein)	102.8 ± 15.3	34.4 ± 12.1*	35.5 ± 4.9*	43.3 ± 4.7* ^,^ ^#^
GPx (Unit^c^/mg protein)	26.8 ± 2.4	20.6 ± 1.6	20.7 ± 3.8	21.3 ± 3.3
SOD (Unit^d^/mg protein)	10.9 ± 1.5	5.9 ± 1.3*	8.9 ± 2.1	13.3 ± 1.4^#^
CAT (Unit^e^/mg protein)	45.6 ± 5.9	20.4 ± 4.7*	16.4 ± 3.2*	24.5 ± 1.6* ^,^ ^#^

*Note:* Data are presented as means ± standard error of the mean (SEM) for five rats per group.

Abbreviations: 8‐OHdG, 8‐hydroxy‐2′‐deoxyguanosine; AOM, azoxymethane; CAT, catalase; DSS, dextran sodium sulfate; GPx, glutathione peroxidase; GSH, reduced glutathione; SOD, superoxide dismutase.

^a^ DRB3 refers to defatted rice bran at a dosage of 3 g per kg body weight of rat.

^b^ DRB6 refers to defatted rice bran at a dosage of 6 g per kg body weight of rat.

^c^ One unit of GPx is defined as the amount of enzyme that catalyzes the oxidation of 1 nmol of NADPH per minute at 25°C.

^d^ One unit of SOD is defined as the amount of enzyme required to achieve 50% dismutation of superoxide.

^e^ One unit of CAT is defined as the amount of enzyme that facilitates the formation of 1.0 nmol of formaldehyde per minute at 25°C.

* *p* value less than 0.05, a statistically significant difference compared to the control group.

^#^ *p* value less than 0.05, a statistically significant difference compared to the AOM/DSS group.

#### 3.6.2 The Effects of DRB on Antioxidant Markers in the Colon of Rats Subjected to AOM/DSS‐Induced Oxidative Stress

The results on the antioxidant markers are presented in Table 2. All groups of rats treated with AOM/DSS exhibited a significantly lower level of GSH than the control group (*p* < 0.05). Notably, the AOM/DSS + DRB6 group demonstrated a significantly higher level of GSH than the AOM/DSS group (*p* < 0.05). The analysis of GPx activity revealed no significant differences among the control group, the AOM/DSS group, the AOM/DSS + DRB3 group, and the AOM/DSS + DRB6 group (*p* ≥ 0.05). The analysis of SOD activity revealed that the AOM/DSS group exhibited a significantly lower level of SOD activity compared to the control group (*p* < 0.05). Conversely, no significant differences in SOD activity were observed between the AOM/DSS + DRB3 and AOM/DSS + DRB6 groups when compared to the control group (*p* ≥ 0.05). Notably, the SOD activity in the AOM/DSS + DRB6 group was significantly higher than that in the AOM/DSS group (*p* < 0.05). In the context of CAT activity, all groups of rats subjected to AOM/DSS treatment demonstrated significantly lower levels of CAT activity when compared to the control group (*p* < 0.05). Notably, the AOM/DSS + DRB6 group exhibited a significantly higher level of CAT activity than the AOM/DSS group (*p* < 0.05).

### 3.7 Microbiota Composition Changes in Rats Induced by AOM/DSS and Supplemented With Defatted Rice Bran

**FIGURE 5:**
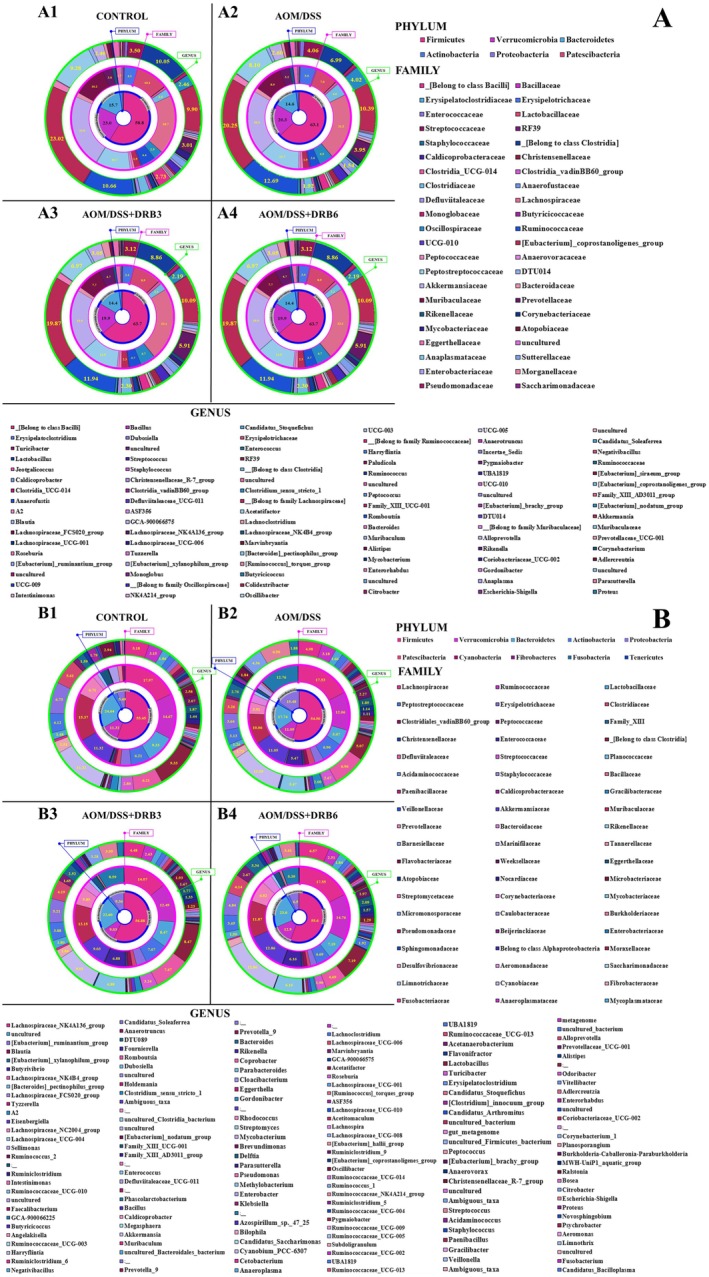
Relative abundance of fecal (A) and mucosal (B) microbiota across experimental groups. The relative abundance of fecal (A) and mucosal (B) microbiota composition at the phylum (inner ring), family (middle ring), and genus (outer ring) levels across the experimental groups: (A1, B1) Control, (A2, B2) AOM/DSS, (A3, B3) AOM/DSS + DRB3, and (A4, B4) AOM/DSS + DRB6. Relative abundance data corresponding to the phylum, family, and genus levels are available in Tables S2–S4.

The composition of fecal microbiota at the phylum level revealed notable shifts following AOM/DSS treatment, as shown in Figure 5A. Firmicutes increased by 7.88%, with further elevations observed after DRB supplementation, particularly at a dosage of 3 g/day, which resulted in a 17.43% increase. Proteobacteria, a marker of dysbiosis, also increased by 8.33%, but this was significantly reduced by DRB3 (−46.15%) and DRB6 (−15.38%). Verrucomicrobia declined after AOM/DSS treatment (−12.03%) and further decreased with DRB3 (−29.22%, Figure 5A3), while DRB6 partially restored its levels (−13.68%, Figure 5A4). Both Bacteroidota and Actinobacteria exhibited reductions under AOM/DSS (Figure 5A2), although there was partial recovery in the DRB groups. Notably, Patescibacteria, which decreased due to AOM/DSS (−14.10%), increased significantly with DRB3 (+30.86%, Figure 5A3) and DRB6 (+31.15%, Figure 5A4).

At the family level, Akkermansiaceae declined under AOM/DSS (−11.99%) and experienced a further reduction with DRB3 (−29.18%). However, DRB6 partially restored its abundance, increasing by 17.89%. In contrast, Lachnospiraceae increased following AOM/DSS (+22.99%), declined with DRB3 (−51.45%), but rebounded with DRB6 (+10.40%). Notably, Lactobacillaceae decreased under AOM/DSS (−30.35%) but recovered with DRB3 (+30.04%) and DRB6 (+26.75%). Ruminococcaceae followed a similar trend, declining with AOM/DSS (−32.66%) but improving under DRB6 (+27.42%). Oscillospiraceae significantly increased with AOM/DSS (+53.42%) but decreased with DRB3 (−56.70%).

At the genus level, *Clostridium sensu stricto 1* increased with AOM/DSS (+63.41%), while *Lactobacillus* (−30.35%) and *Akkermansia* (−12.04%) exhibited declines. The DRB3 and DRB6 treatments modulated these changes differently; *Akkermansia* continued to decrease in the DRB3 group (−19.56%) but showed partial recovery in the DRB6 group (+17.89%). *Lachnospiraceae_NK4A136* increased in both DRB groups, suggesting a potential prebiotic effect.

For mucosal microbiota composition (Figure 5B), AOM/DSS (Figure 5B2) induced significant shifts in the mucosal microbiota, with the phylum of Proteobacteria exhibiting a remarkable increase of 181.96%. This increase was significantly reduced by DRB3 (−39.55%, Figure 5B3) and DRB6 (−58.01%, Figure 5B4). Bacteroidota experienced a decline of 27.98% but partially recovered with DRB3 (+27.41%) and DRB6 (+29.44%). Verrucomicrobia remained stable following AOM/DSS but declined with DRB3 (−20.23%), while DRB6 increased its abundance by 13.61%. Actinobacteria dropped sharply by 60.87%, but DRB3 (+69.23%) and DRB6 (+68.38%) restored their levels.

At the family level, Muribaculaceae was significantly reduced by AOM/DSS (−34.55%) but was partially restored by DRB3 (+30.86%) and DRB6 (+17.99%). Ruminococcaceae (−17.80%) and Lactobacillaceae (−45.65%) exhibited a similar pattern, showing notable recovery with DRB supplementation. Enterobacteriaceae experienced a dramatic increase (+14,078%) following AOM/DSS treatment, with DRB3 (−32.66%) and DRB6 (−57.75%) mitigating this effect.

At the genus level, both *Lactobacillus* and an unclassified genus within the Muribaculaceae family exhibited a decline under AOM/DSS treatment, with decreases of 45.69% and 45.99%, respectively. However, the administration of DRB3 (Figure 5B3) and DRB6 (Figure 5B4) significantly restored these genera, resulting in an increase of 67.22% for *Lactobacillus* with DRB3 and 41.85% with DRB6. *Akkermansia* experienced a decline of 18.25% under DRB3 but showed an increase of 13.57% with DRB6. *Alloprevotella* displayed a similar trend, decreasing following AOM/DSS treatment but recovering with DRB supplementation.

### 3.8 Network Topology Reveals Key Microbial Hubs and Interaction Patterns

**FIGURE 6:**
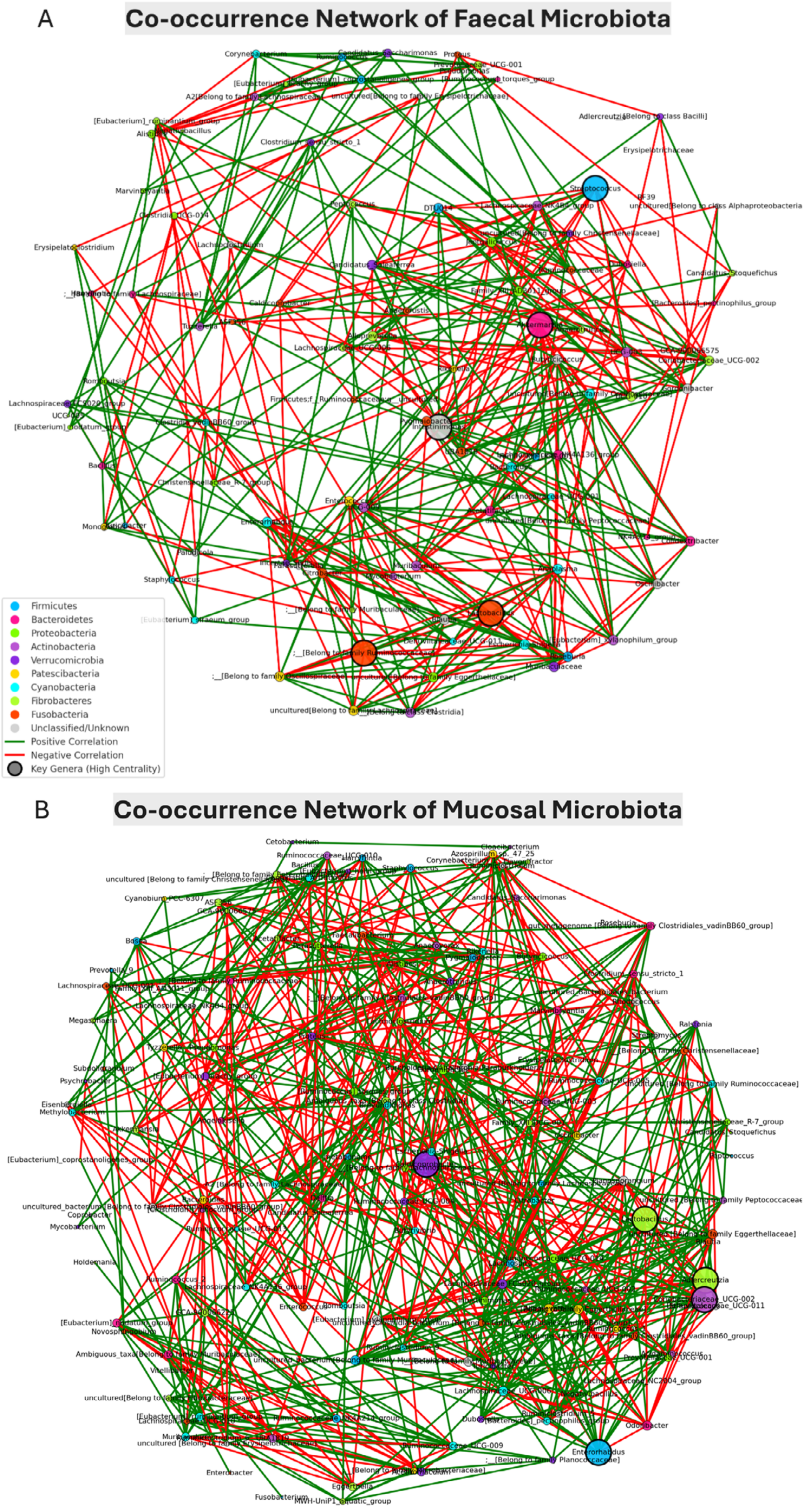
Co‐occurrence network of bacterial genera in fecal and mucosal samples across experimental groups. The co‐occurrence network illustrates significant correlations (*p* < 0.05) among bacterial genera in fecal (A) and mucosal (B) samples based on genus‐level relative abundance data from four experimental groups: Control, AOM/DSS, AOM/DSS + DRB3 (defatted rice bran 3 g), and AOM/DSS + DRB6 (defatted rice bran 6 g). Nodes represent individual bacterial genera, with node size proportional to degree centrality, indicating the relative importance of each genus within the microbial network. Key genera with high centrality values are highlighted with bold black borders and larger node sizes to emphasize their pivotal roles in maintaining network structure and stability. Node colors correspond to bacterial phyla: Firmicutes (neon blue), Bacteroidetes (deep pink), Proteobacteria (chartreuse), Actinobacteria (medium orchid), Verrucomicrobia (blue‐violet), Patescibacteria (bright gold), Cyanobacteria (cyan), Fibrobacteres (green‐yellow), Fusobacteria (orange‐red), and Unclassified/Unknown (light gray). Edges represent significant correlations between genera: Green edges indicate positive correlations (co‐occurrence or mutualistic relationships). Red edges indicate negative correlations (competitive or inhibitory interactions). Edge thickness reflects the correlation strength, with thicker lines representing stronger relationships.

The co‐occurrence network analysis of bacterial genera from fecal and mucosal samples reveals intricate microbial interactions under varying experimental conditions (Figure 6). Nodes represent bacterial genera, with edges denoting statistically significant correlations (*p* < 0.05). The size of the nodes corresponds to their centrality, with larger nodes indicating a more substantial influence on microbial interactions. Green edges represent positive associations, suggesting potential mutualism, while red edges signify negative correlations indicative of competitive interactions. The thickness of the edges corresponds to the strength of the associations.

In the fecal microbiota network (Figure 6A), *Akkermansia*, *Intestinimonas*, *Lactobacillus*, *Streptococcus*, and an uncultured genus belonging to the Ruminococcaceae family emerged as key hubs with high centrality, underscoring their pivotal role in maintaining microbial stability. These genera exhibited extensive interconnections, highlighting their ecological significance. Compared to the control, enhanced network connectivity in the AOM/DSS + DRB groups suggests that DRB supplementation may promote microbial interactions and gut homeostasis. Notably, the increased centrality of *Akkermansia* in DRB‐supplemented groups supports its role in maintaining gut barrier integrity and modulating the immune response.

In the mucosal microbiota network (Figure 6B), *Eubacterium ruminantium* , *Bacteroides pectinophilus* , and *Clostridium innocuum* exhibited the highest centrality, indicating their significance in the mucosal microbial community. The AOM/DSS + DRB group demonstrated increased microbial connectivity, particularly among beneficial genera, suggesting dietary intervention may enhance microbial resilience. Predominantly positive correlations within the same phylum and negative correlations between different phyla underscore the complex ecological and competitive dynamics that shape microbial community structure.

### 3.9 Correlation Patterns and Co‐Occurrence Dynamics

**FIGURE 7:**
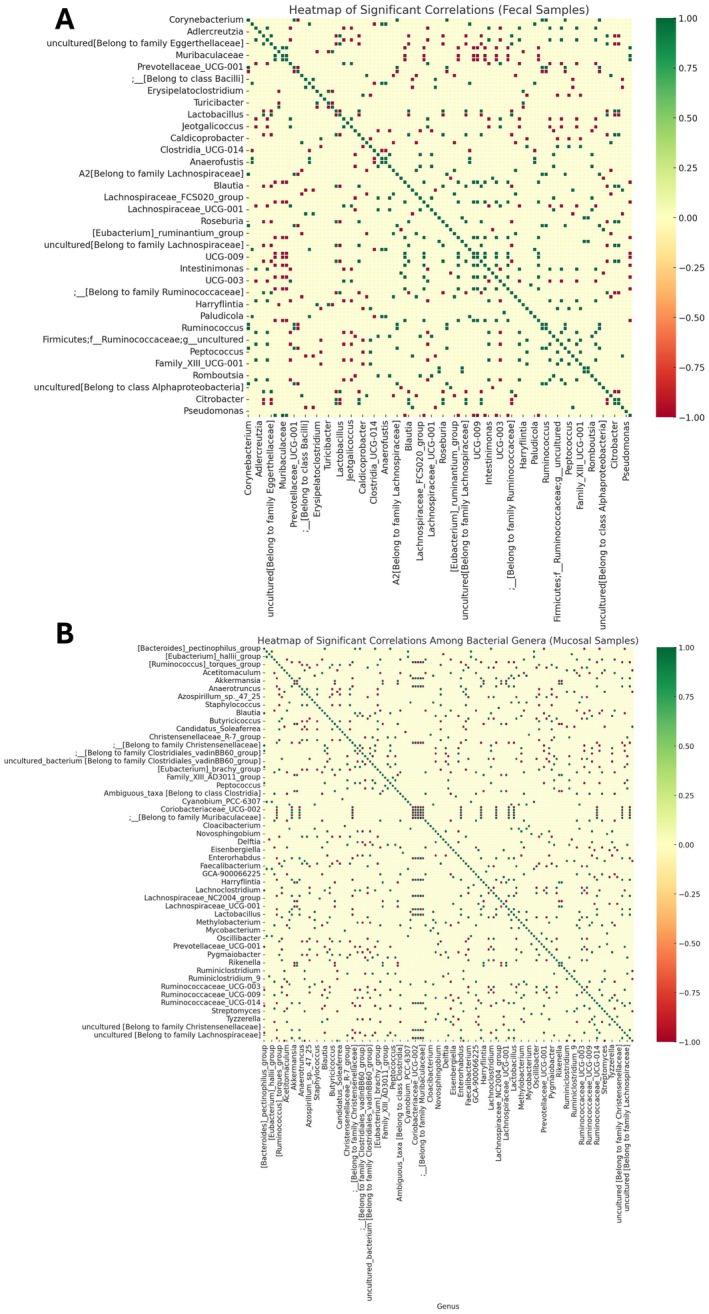
Heatmap of significant correlations among bacterial genera in fecal (A) and mucosal (B) samples. The heatmap displays significant correlations (*p* < 0.05) among bacterial genera based on genus‐level relative abundance data from four experimental groups: Control, AOM/DSS, AOM/DSS + DRB3 (defatted rice bran 3 g), and AOM/DSS + DRB6 (defatted rice bran 6 g). Spearman's correlation coefficients were calculated to assess pairwise associations, and only statistically significant correlations were visualized. The color gradient represents the strength and direction of the correlations: Green shades indicate *positive correlations*, suggesting potential mutualistic or co‐occurrence relationships between genera. Red shades represent *negative correlations*, indicating potential competitive or inhibitory interactions. The intensity of the colors reflects the correlation strength, with more intense colors corresponding to stronger associations. Genera with higher connectivity are clustered centrally, reflecting potential hub roles within the microbial network. This heatmap highlights key microbial interactions that dietary interventions may influence.

Based on relative abundance data, genus‐level co‐occurrence networks were analyzed to assess microbial interactions in response to defatted rice bran (DRB) supplementation. Spearman's correlation analysis identified significant associations (*p* < 0.05), visualized as heatmaps (Figure 7A,B). Positive correlations (green) suggest cooperative interactions, while negative correlations (red) indicate competition.

#### 3.9.1 Fecal Microbial Correlation Patterns

The fecal microbiota network (Figure 7A) revealed strong positive correlations between Firmicutes and Bacteroidota, suggesting functional complementarity. Negative correlations between different phyla indicate niche differentiation. Key genera, including *Akkermansia, Intestinimonas, Lactobacillus*, and unclassified genera within the Ruminococcaceae family, exhibited high connectivity, underscoring their significant ecological roles. DRB supplementation enhanced microbial interactions, stabilizing gut microbiota composition. The AOM/DSS treatment disrupted microbial correlations, increasing negative associations, whereas DRB partially restored beneficial interactions, particularly within Lachnospiraceae and Oscillospiraceae.

#### 3.9.2 Mucosal Microbial Correlation Patterns

The mucosal microbiota network (Figure 7B) showed strong positive correlations between Firmicutes and Bacteroidota, particularly between *Eubacterium ruminantium* and *Bacteroides pectinophilus* , suggesting key microbial hubs. Conversely, negative correlations between Proteobacteria and Firmicutes highlight competitive interactions, particularly involving *Pseudomonas* and *Citrobacter*. Increased microbial connectivity in the AOM/DSS + DRB groups suggests that DRB supplementation enhances cooperative interactions, potentially improving gut barrier integrity and reducing inflammation.

### 3.10 Identification of Key Genera Based on Network‐Centrality Metrics

Network‐centrality analysis identified key bacterial genera that shape microbial communities. Degree, betweenness, and closeness centrality metrics were employed to evaluate their influence.

#### 3.10.1 Key Genera in Fecal Microbiota

*Akkermansia*, *Intestinimonas*, *Lactobacillus*, *Streptococcus*, and uncultured genera within Ruminococcaceae taxa demonstrated the highest centrality, functioning as microbial hubs (Table S5). The low betweenness centrality indicates a distributed network structure. The increased centrality of beneficial genera in the AOM/DSS + DRB groups suggests that DRB supplementation enhances microbial stability and interactions, potentially aiding in the prevention of microbial dysbiosis.

#### 3.10.2 Key Genera in Mucosal Microbiota

*Coriobacteriaceae_UCG‐002*, *Adlercreutzia*, *Lactobacillus*, *Caldicoprobacter*, and *Enterorhabdus* exhibited high centrality, indicating their significant role in maintaining mucosal stability (Table S6). The mucosal networks demonstrated strong intraphylum correlations, which suggest functional interactions among the taxa. The low betweenness centrality indicates that key taxa primarily function as local hubs within the network. The increased centrality of *Lactobacillus* and *Adlercreutzia* in the AOM/DSS + DRB groups suggests that DRB supplementation enhances beneficial microbial interactions, thereby improving gut barrier function and regulating inflammation.

### 3.11 Changes in Cecal Short‐Chain Fatty Acids in AOM/DSS‐Induced Rats Supplemented With Defatted Rice Bran

**FIGURE 8:**
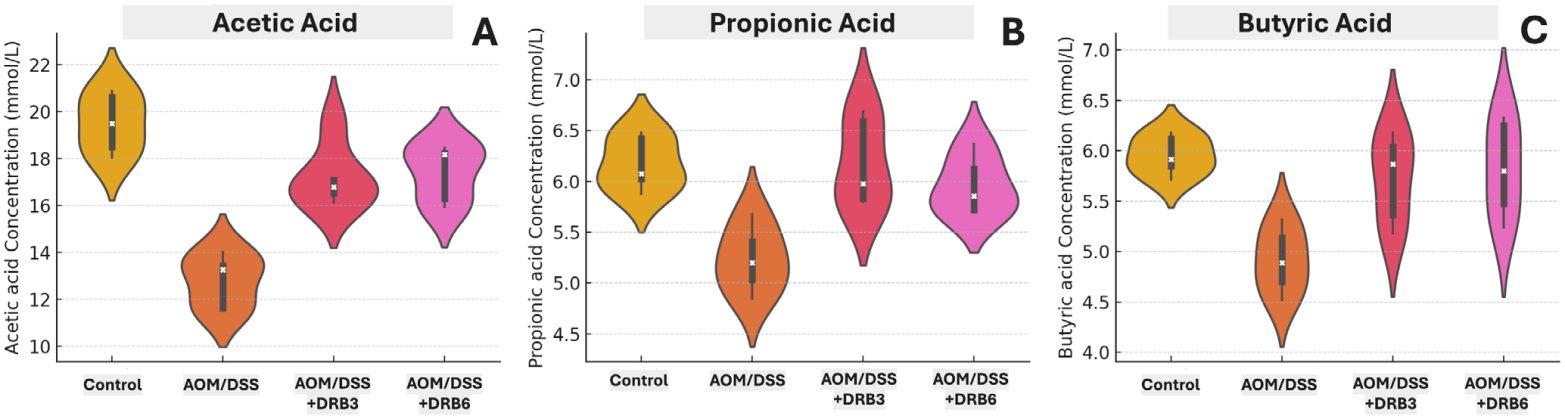
Violin plots depicting the distribution of short‐chain fatty acid (SCFA) concentrations across experimental groups. Violin plots illustrate the variability of acetic acid (A), propionic acid (B), and butyric acid (C) concentrations in Control, AOM/DSS, AOM/DSS + DRB3, and AOM/DSS + DRB6 groups. The white dot represents the median, the thick central bar indicates the interquartile range, and the thin line represents the full data distribution.

The effects of AOM/DSS treatment and defatted rice bran (DRB) supplementation on cecal short‐chain fatty acids (SCFAs) were evaluated using violin plots (Figure 8), illustrating SCFA distribution and variability across experimental groups.

The control group exhibited the highest acetic acid concentrations (Figure 8A), while AOM/DSS treatment significantly reduced levels, indicating metabolic disturbances linked to inflammation and dysbiosis. DRB supplementation, particularly at a higher dose (DRB6), restored acetic acid levels in a dose‐dependent manner. A similar trend was observed for propionic acid (Figure 8B) and butyric acid (Figure 8C), where AOM/DSS‐induced depletion was mitigated by DRB, with DRB6 restoring butyric acid levels to near‐control values, underscoring its potential prebiotic effects.

**FIGURE 9:**
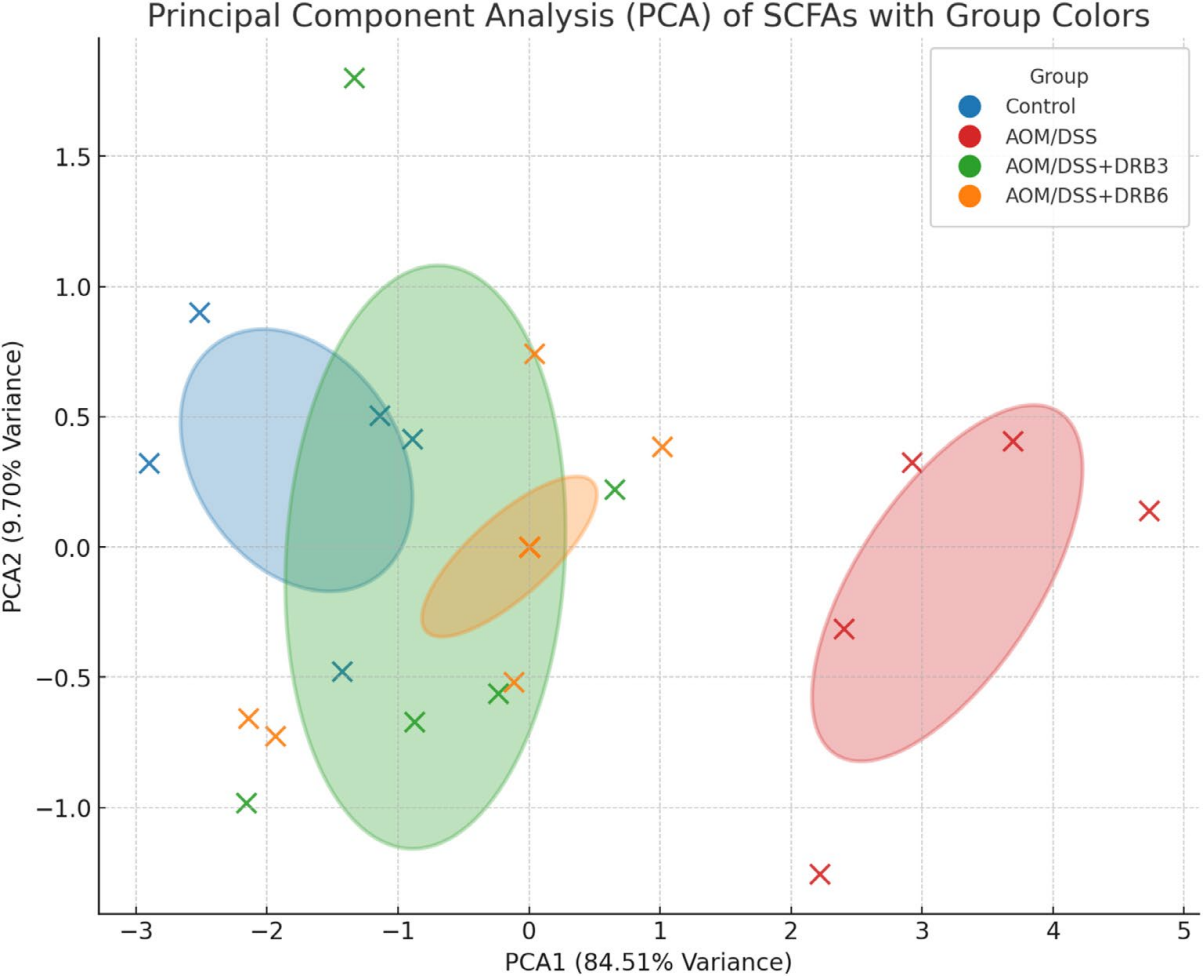
Principal Component Analysis (PCA) of SCFA profiles. PCA plot depicting the clustering of samples based on their SCFA composition. Each group is color‐coded: Control (blue), AOM/DSS (red), AOM/DSS + DRB3 (green), and AOM/DSS + DRB6 (orange). PCA1 (84.51%) and PCA2 (9.70%) explain the majority of the variance, illustrating distinct metabolic shifts between groups.

Principal Component Analysis (PCA) (Figure 9) further elucidates the metabolic alterations of SCFAs. PCA1, which accounts for 84.51% of the variance, effectively distinguishes between the control and AOM/DSS groups, highlighting inflammation‐induced metabolic disruptions. Notably, DRB supplementation partially restored SCFA metabolism, with samples from the DRB6 group clustering closer to the control group than those from the DRB3 group, indicating a more pronounced modulatory effect. PCA2, which explains 9.70% of the variance, captures inter‐individual variability within the treatment groups, reflecting subtle metabolic differences among individuals receiving the same intervention. These findings confirm the disruption of SCFA metabolism in AOM/DSS‐induced colorectal carcinogenesis and demonstrate the potential of DRB, particularly at a dosage of 6 g/day, to restore SCFA profiles and promote metabolic health by modulating microbial fermentation processes.

### 3.12 Correlations Between Intestinal Microbiota and Redox‐Associated Biomarkers: A Spearman Correlation Analysis

To clarify the relationship between gut microbiota and redox homeostasis, Spearman's rank correlation coefficients were calculated between microbiota composition (phyla, families, and genera) from fecal and mucosal samples and biochemical markers, including SCFAs (acetic acid, propionic acid, and butyric acid), oxidative stress indicators (8‐OHdG and protein carbonyl), and antioxidant biomarkers (GSH, GPx, SOD, and CAT). Correlation strength was categorized as strong (|*r*| > 0.6) or moderate (|*r*| = 0.4–0.6).

**FIGURE 10:**
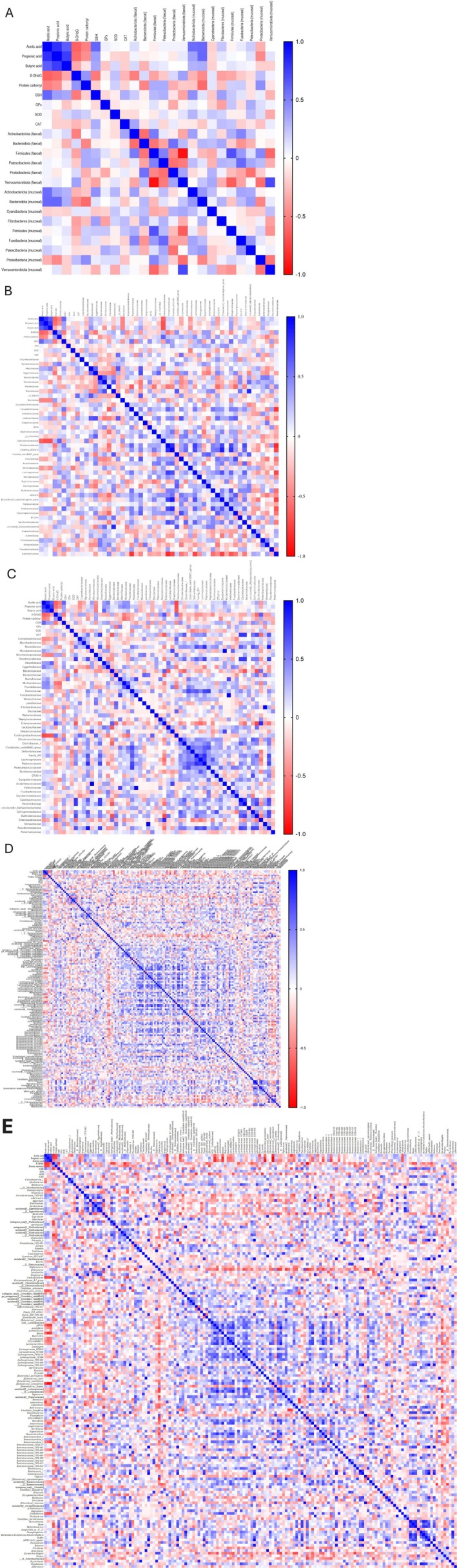
Correlation analysis of intestinal microbiota with short‐chain fatty acids (SCFAs) and antioxidative parameters. The Spearman's correlation coefficient (*r*) was used to assess the relationships among the abundance of intestinal microbiota at the phylum level (A), the family level of fecal microbiota (B), the family level of mucosal microbiota (C), the genera of fecal microbiota (D), and the genera of mucosal microbiota (E); short‐chain fatty acids (SCFAs), specifically acetic acid, propionic acid, and butyric acid; and antioxidative parameters, including 8‐hydroxy‐2′‐deoxyguanosine (8‐OHdG), protein carbonyl, reduced glutathione (GSH), glutathione peroxidase (GPx), superoxide dismutase (SOD), and catalase (CAT). Spearman's correlation coefficient varies from +1 to −1, with 0 signifying the absence of correlation. Values above ±0.60 to ±1 indicate a strong correlation, those ranging from ±0.40 to ±0.60 represent a moderate correlation, while values below ±0.40 are considered weak correlations. Negative correlations are shown in red, whereas positive correlations are shown in blue, with the color intensity proportional to the correlation strength.

No significant connections were detected at the phylum level (Figure 10A). Moderate associations were observed: fecal Actinobacteriota exhibited a negative correlation with the oxidative DNA damage marker 8‐OHdG (*r* = −0.504, *p* < 0.05), while Bacteroidota showed a negative correlation with protein carbonyl levels (*r* = −0.484, *p* < 0.05), suggesting a potential role of these phyla in the modulation of oxidative stress. Mucosal Actinobacteriota (*r* = 0.574, *p* < 0.01) and Bacteroidota (*r* = 0.589, *p* < 0.01) exhibited a positive correlation with acetic acid, indicating a contributing involvement in mucosal SCFA metabolism.

At the familial level (Figure 10B), notable fecal associations comprised substantial negative correlations between the abundance of Caldicoprobacteraceae and the concentrations of acetic acid (*r* = −0.622, *p* < 0.01), propionic acid (*r* = −0.684, *p* < 0.001), and butyric acid (*r* = −0.684, *p* < 0.001), as well as a strong positive correlation with the oxidative stress marker protein carbonyl (*r* = 0.611, *p* < 0.01). The data suggest that Caldicoprobacteraceae may negatively impact intestinal short‐chain fatty acid (SCFA) synthesis and increase oxidative stress. Analysis of mucosal microbiota demonstrated significant family‐level associations. The abundance of Streptomycetaceae shows a robust positive connection with CAT activity (*r* = 0.763, *p* < 0.001), suggesting a potential improvement of antioxidant capability. Atopobiaceae exhibited a positive correlation with butyric acid (*r* = 0.613, *p* < 0.01), but Bacteroidaceae demonstrated an inverse correlation with CAT (*r* = −0.665, *p* < 0.001). Furthermore, Enterococcaceae exhibited dual correlations, positively correlating with 8‐OHdG (*r* = 0.665, *p* < 0.001) and negatively with CAT (*r* = −0.647, *p* < 0.01), indicating its involvement in intensifying oxidative stress and undermining antioxidant defenses.

At the genus level, several significant correlations were identified between specific gut microbiota and redox‐associated biomarkers in both fecal and mucosal samples. In fecal samples (Figure 10C), *Streptomyces* showed a strong positive correlation with CAT activity (*r* = 0.641, *p* < 0.01), indicating a potential role in enhancing antioxidant‐enzyme defense. Likewise, *Coriobacteriaceae_UCG‐002* exhibited a robust positive correlation with butyric acid (*r* = 0.613, *p* < 0.01), suggesting its contribution to SCFA biosynthesis. In contrast, *Enterococcus* was negatively correlated with CAT activity (*r* = −0.619, *p* < 0.01), implying an inhibitory influence on antioxidant responses. Moreover, *Caldicoprobacter* showed strong negative correlations with acetic acid (*r* = −0.679, *p* < 0.001) and propionic acid (*r* = −0.641, *p* < 0.01), suggesting potential adverse effects on SCFA metabolism.

In mucosal samples (Figure 10D), a similar pattern of associations was observed. *Coriobacteriaceae_UCG‐002* again demonstrated a positive correlation with mucosal butyric acid levels (*r* = 0.613, *p* < 0.01). Several genera were positively associated with CAT activity, including *Butyricicoccus* (*r* = 0.774, *p* < 0.001), *Brevundimonas* (*r* = 0.704, *p* < 0.001), *Bosea* (*r* = 0.692, *p* < 0.001), *Ruminococcaceae_UCG‐010* (*r* = 0.669, *p* < 0.001), *Methylobacterium* (*r* = 0.660, *p* < 0.01), *Streptomyces* (*r* = 0.641, *p* < 0.01), and *Pseudomonas* (*r* = 0.632, *p* < 0.01). These associations suggest a broad microbial contribution to host antioxidative capacity. On the other hand, several mucosal genera exhibited negative associations with SCFAs and antioxidant markers. *Caldicoprobacter* was negatively correlated with all three major SCFAs—acetic (*r* = −0.679, *p* < 0.001), propionic (*r* = −0.641, *p* < 0.01), and butyric acid (*r* = −0.608, *p* < 0.01)—indicating disruption of microbial fermentation processes. Similarly, *Roseburia* showed a negative correlation with acetic acid (*r* = −0.617, *p* < 0.01), while *[Bacteroides]_pectinophilus* and *[Eubacterium]_xylanophilum* were inversely associated with butyric (*r* = −0.715, *p* < 0.001) and propionic acid (*r* = −0.669, *p* < 0.01), respectively. *Anaerotruncus* was negatively correlated with both acetic acid (*r* = −0.609, *p* < 0.01) and GSH levels (*r* = −0.641, *p* < 0.01), suggesting redox dysregulation. Furthermore, *Tyzzerella* showed a negative correlation with SOD activity (*r* = −0.616, *p* < 0.01), whereas *Proteus* demonstrated a positive association with SOD (*r* = 0.607, *p* < 0.01). Notably, *Enterococcus* remained negatively correlated with CAT activity in mucosal samples (*r* = −0.619, *p* < 0.01), consistent with its fecal profile.

Among the short‐chain fatty acids, acetic and propionic acids demonstrated a robust positive association (*r* = 0.815, *p* < 0.001), indicating substantial co‐production or a common microbial source. Acetic acid exhibits a positive correlation with butyric acid (*r* = 0.639, *p* < 0.01), indicating synchronized metabolic pathways among SCFAs. Acetic acid exhibited a negative correlation with 8‐OHdG (*r* = −0.627, *p* < 0.01) and a positive correlation with GSH (*r* = 0.602, *p* < 0.01), highlighting its possible antioxidative function. Ultimately, propionic acid exhibited a robust association with butyric acid (*r* = 0.759, *p* < 0.001). However, no other statistically strong correlations were observed between propionic or butyric acids and the other assessed redox‐related indicators.

## 4 Discussion

The current study demonstrates that defatted rice bran (DRB) is a nutrient‐rich by‐product with substantial potential as a functional dietary ingredient, highlighting its antioxidant, anti‐inflammatory, and prebiotic properties. Our comprehensive analyses revealed high levels of dietary fiber, phenolic compounds, flavonoids, and phytic acid, all of which contribute synergistically to its biological activities.

DRB serves as a substantial source of dietary fiber, comprising both insoluble dietary fiber (IDF) and soluble dietary fiber (SDF). This study reported a total dietary fiber (TDF) content of 28.3 g per 100 g dry weight (DW), comparable to findings by Daou and Zhang (2014), who reported 32.9 ± 0.3 g per 100 g DW. Notably, the majority of the fiber fraction in DRB was IDF (21.8 g/100 g DW), constituting approximately 93.8% of the TDF and composed predominantly of hemicellulose (54.5%), cellulose (33.4%), and lignin (5.8%). These structural polysaccharides confer specific physicochemical properties, such as water‐holding capacity, fecal bulking, and intestinal transit acceleration, which contribute to gastrointestinal health (Blackwood et al. 2000). Compared to conventional rice bran, which contains 23.3 g TDF, 21.2 g IDF, and 2.2 g SDF per 100 g DW (Faria et al. 2012; Park et al. 2017), DRB presents higher concentrations of both IDF and SDF, highlighting its functional superiority. The IDF components, particularly cellulose and hemicellulose, escape enzymatic digestion in the small intestine and reach the colon, where they undergo partial microbial fermentation. Although less fermentable than SDF, IDF still supports microbial diversity and contributes to SCFA production—especially acetic and butyric acids—via cross‐feeding mechanisms (Anderson et al. 2009). In addition, IDF plays a key role in mechanical gut stimulation and the maintenance of colonic health by reducing luminal transit time, lowering intraluminal pressure, and adsorbing potential carcinogens and bile acids, thereby limiting their contact with the colonic epithelium (Anderson et al. 2009). Lignin, the least fermentable IDF component, contributes antioxidant properties by binding ROS and modulating microbial metabolites (Das et al. 2023).

In addition to its high insoluble fiber content, DRB contains appreciable levels of soluble dietary fiber (SDF), accounting for approximately 6.5 g per 100 g DW in this study. SDF fractions in rice bran typically include arabinoxylans, β‐glucans, pectins, and small amounts of resistant starch, which exhibit significant biological activity. These SDF components possess high water‐holding capacity and viscosity, forming gels in the gastrointestinal tract that modulate nutrient absorption and bile acid reabsorption, ultimately aiding in lipid regulation and cholesterol reduction (Anderson et al. 2009; Beloshapka et al. 2016).

Soluble dietary fiber (SDF) is a highly fermentable substrate for the colonic microbiota, which promotes the growth of beneficial bacteria such as *Bifidobacterium*, *Lactobacillus*, and *Akkermansia*. This microbial fermentation produces short‐chain fatty acids (SCFAs), specifically butyrate, acetate, and propionate, which are important in intestinal health. Butyrate is a key energy source for colonocytes, increases mucosal barrier integrity, and has potent anti‐inflammatory and anti‐carcinogenic effects by regulating NF‐κB and HDAC pathways (Tedelind et al. 2007; Venegas et al. 2019). Propionate and acetate help maintain immunological homeostasis and redox signaling, which strengthen the antioxidative defense system (González‐Bosch et al. 2021).

Soluble dietary fiber (SDF), especially arabinoxylans and β‐glucans, has been associated with reduced expression of proinflammatory cytokines, such as TNF‐α and IL‐6, and oxidative stress markers in both in vitro and in vivo studies. These fibers enhance SCFA‐mediated signaling via G‐protein‐coupled receptors, promoting regulatory T‐cell expansion and suppressing inflammation‐linked tumorigenesis (Moerings et al. 2023; Singh and Bhardwaj 2023). Moreover, pectins, another component of SDF, have been shown to inhibit the growth of colon cancer cells and support gut barrier function by increasing mucin production and modulating epithelial gene expression (Khorasaniha et al. 2023).

Together, these properties position the SDF in DRB as not only fermentable substrates but also bioactive agents capable of orchestrating complex interactions between host immunity, oxidative stress regulation, microbial ecology, and cancer prevention. Thus, the inclusion of DRB as a dietary intervention may provide synergistic protective effects through the actions of both insoluble and soluble fiber fractions (Anderson et al. 2009; Daou and Zhang 2014; Zheng et al. 2024).

Importantly, DRB contains an abundance of phenolic compounds, flavonoids, and phytic acid, which contribute synergistically to its potent antioxidant and anti‐inflammatory properties. Quantitative analysis in this study revealed that DRB provided 312.0 ± 0.4 mg GAE/100 g dry weight (DW) of total phenolic content (TPC), 10.9 ± 0.2 mg QE/100 g DW of total flavonoid content (TFC), and 8.0 ± 0.1 g/100 g DW of phytic acid. These values are slightly lower than those reported in earlier studies, likely due to degradation during industrial oil extraction, which involves elevated temperatures and solvent exposure (Min et al. 2014; Scaglioni et al. 2014). Despite some losses, DRB retains substantial bound phenolics embedded in its fiber matrix. A portion of these compounds is released in the colon through microbial fermentation, enabling targeted antioxidant and anti‐inflammatory effects at the site of action (Adom and Liu 2002; Guo and Beta 2013; Zhao et al. 2018; Zheng et al. 2024).

The antioxidant capacity of DRB was further confirmed in this study by using three complementary in vitro assays, DPPH, FRAP, and ORAC assays, each targeting a distinct mechanism of antioxidant action. The DPPH assay quantifies free radical‐scavenging activity by measuring the ability of antioxidants to donate hydrogen atoms and neutralize DPPH• radicals. The FRAP assay assesses reducing power based on the capacity to convert ferric (Fe^3+^) to ferrous (Fe^2+^) ions under acidic conditions. The ORAC assay measures peroxyl radical inhibition, which is particularly relevant to lipid peroxidation and biological oxidative stress. The combined application of these methods adheres to current scientific consensus, which suggests that no single assay can fully characterize the antioxidant capacity of complex food matrices. Each assay targets different oxidative pathways or reactive species, and their integration yields a more robust and biologically meaningful assessment (Munteanu and Apetrei 2021; Sadowska‐Bartosz and Bartosz 2022).

To complement the antioxidant assays, the phenolic acid composition of the DRB used in this study was previously analyzed using high‐performance liquid chromatography (HPLC), with ferulic acid (76.7 ± 0.6 mg/100 g DW) and p‐coumaric acid (10.8 ± 0.2 mg/100 g DW) identified as predominant constituents, alongside syringic acid, vanillic acid, and other bound phenolics (Tajasuwan et al. 2022). These compounds are recognized for their roles in modulating oxidative stress and inflammation, supporting the mechanistic basis for DRB's observed bioactivity in the present model.

Specifically, phenolic acids and flavonoids neutralize reactive oxygen species (ROS), chelate redox‐active metals, and inhibit stress‐related signaling pathways. Phytic acid further enhances antioxidant protection by limiting hydroxyl radical formation via metal chelation, thereby reducing lipid peroxidation (Graf and Eaton 1990; Spiegel et al. 2020; Tarahovsky et al. 2008). Collectively, these bioactives contribute not only to redox balance but also to immune regulation and epithelial barrier preservation, underscoring the multifaceted protective capacity of DRB (Goufo and Trindade 2014; Graf and Eaton 1990).

Simulated gastrointestinal digestion revealed considerable bioaccessibility of total phenolic content (TPC) and total flavonoid content (TFC) in DRB, confirming previous reports on the gastrointestinal stability and release of rice bran‐derived phenolics (Nignpense et al. 2022). The partial liberation of bound phenolic compounds under digestive conditions enhances their availability for intestinal absorption and interaction with gut epithelial cells, supporting their biological relevance.

Although porcine‐derived enzymes were used in this in vitro model to simulate human digestion, in accordance with standardized INFOGEST protocols (Brodkorb et al. 2019), their catalytic activity and substrate specificity differ slightly from their human counterparts. However, comparative studies suggest that these differences are minor and unlikely to significantly alter the relative trends in phenolic bioaccessibility. Nonetheless, this methodological constraint should be acknowledged when extrapolating in vitro findings to human physiological contexts (Kageyama et al. 2010; Marengo et al. 2022).

Subsequent functional assays using Caco‐2 cells demonstrated that digested DRB significantly attenuated intracellular ROS accumulation and reduced key proinflammatory cytokines, including interleukin‐6 (IL‐6), interleukin‐8 (IL‐8), and tumor necrosis factor‐alpha (TNF‐α). These cytokines play central roles in the pathogenesis of inflammatory bowel disease (IBD) and colorectal cancer (CRC), and their suppression indicates a marked anti‐inflammatory effect of DRB in the gut epithelium (Kaval et al. 2022; Soufli et al. 2016; Wei et al. 2023).

In addition to polyphenols, flavonoids, and phytic acid, DRB's protective effects may be attributed in part to its bioactive protein hydrolysates and peptides, which are rich in hydrophobic amino acids and have been shown to scavenge free radicals, chelate metal ions, and modulate inflammatory signaling pathways such as NF‐κB and MAPK (Hernández‐Ledesma et al. 2005; Saisavoey et al. 2016; Zou et al. 2016).

In vivo studies showed that DRB significantly lowered AOM/DSS‐induced oxidative stress by lowering DNA (8‐OHdG) and protein (carbonyl) damage while restoring important antioxidant defenses (GSH, SOD, GPx, and CAT). 8‐OHdG represents ROS‐induced DNA oxidation, and protein carbonyls indicate irreversible protein damage. Their simultaneous reduction shows widespread cellular protection crucial in colitis‐associated colorectal carcinogenesis (Ahmed et al. 2024; Bardelčíková et al. 2023; Dzhalilova et al. 2023).

In comparison to other dietary or pharmacological interventions in comparable models, the antioxidative efficacy of DRB appears to be robust. Miyamoto et al. (2008) reported that dietary supplementation with 0.05% 4′‐geranyloxy‐ferulic acid (GAP) significantly attenuated colonic 8‐OHdG levels and tumor development in AOM/DSS‐treated mice. Similarly, Cid‐Gallegos et al. (2020) demonstrated that a 10%–20% cooked chickpea diet effectively decreased protein carbonyl content in a chemically induced colorectal cancer model. Furthermore, DRB's redox‐modulating capacity is of comparable magnitude to that observed with pharmacological agents such as pentoxifylline, which has been shown to restore antioxidant balance and reduce oxidative stress in AOM/DSS‐induced rats (Shirakami et al. 2018).

Previous studies revealed that ferulic acid, one of the predominant phenolic acids in rice bran, has been shown to activate the nuclear factor erythroid 2‐related factor (Nrf2)/antioxidant response element (ARE) pathway, thereby enhancing cellular antioxidant defenses (Kumar et al. 2023; Ma 2013). Furthermore, phytic acid in DRB activates the Nrf2/ARE pathway and suppresses NF‐κB, thereby strengthening endogenous antioxidant defenses (Hou et al. 2022). Although its high affinity for Fe, Zn, and Ca could theoretically limit mineral absorption, evidence suggests the risk is minimal at dietary intakes. Human studies and controlled rodent trials show negligible effects on micronutrient status when phytic acid is consumed within a varied diet (Hurrell et al. 2003; Lopez et al. 2002); routine monitoring may be prudent only in populations already at risk of deficiency.

Taken together, these findings suggest that the combined actions of DRB's phenolic acids, flavonoids, phytic acid, protein‐derived peptides, and dietary fiber fractions offer synergistic protection against oxidative stress and inflammation in both intestinal epithelial and in vivo CRC models, underscoring DRB's potential as a multifunctional ingredient for gastrointestinal health promotion and disease prevention.

The present study also demonstrated that AOM/DSS‐induced colorectal oxidative stress profoundly disrupted gut microbial homeostasis, consistent with previous reports of chemically induced dysbiosis (Li, Peng, Ding, et al. 2023). Notably, the relative abundance of Proteobacteria, a phylum widely recognized as a hallmark of dysbiosis and intestinal inflammation, was markedly elevated in both the fecal and mucosal microbiotas following AOM/DSS treatment (Litvak et al. 2017). Concurrently, several beneficial phyla, including Bacteroidota, Actinobacteria, and Verrucomicrobia, were significantly depleted, reflecting compromised mucosal barrier function, reduced short‐chain fatty acid (SCFA) production, and an overall proinflammatory microbial shift (Li, Peng, Ding, et al. 2023; Lopetuso et al. 2013).

Dietary supplementation with DRB, particularly at a dose of 6 g/kg body weight, effectively ameliorated these microbial imbalances. DRB significantly reduced the abundance of Proteobacteria and its associated family Enterobacteriaceae, both implicated in epithelial damage and genotoxicity (Li, Peng, Ding, et al. 2023; Litvak et al. 2017). At the same time, DRB restored the abundance of several health‐promoting taxa. Notably, the phyla Actinobacteria and Patescibacteria, known for their fermentative capabilities and mucosal support, were re‐established alongside *Streptomyces*, a genus recognized for producing a broad spectrum of bioactive metabolites with antibacterial, antifungal, antiviral, and anticancer properties (Donald et al. 2022).

In addition, the restoration of Verrucomicrobia, particularly Akkermansiaceae and its genus *Akkermansia*, suggests enhanced mucin dynamics and epithelial integrity, reinforcing the mucosal barrier (Everard et al. 2013; Rodríguez‐Daza and de Vos 2023). DRB also reversed the decline of Bacteroidota and associated families, such as Muribaculaceae and Prevotellaceae, including Alloprevotella, which are linked to anti‐inflammatory activity and protection against metabolic disorders and colorectal cancer (Kadhim et al. 2025; Lopetuso et al. 2013; Zhu et al. 2024).

At the genus level, DRB increased key SCFA‐producing and immunoregulatory taxa, including *Lactobacillus* (Lactobacillaceae) and members of the Ruminococcaceae family, both belonging to the Firmicutes phylum. These commensals are pivotal for butyrate production and epithelial immune regulation (Hodgkinson et al. 2023; Li, Peng, Xiao, et al. 2023). Moreover, the observed enrichment of *Lachnospiraceae_NK4A136*, a genus within the Lachnospiraceae family, indicates that DRB may function as a prebiotic substrate, selectively enriching bacterial groups with known anti‐inflammatory and gut‐protective properties (Davani‐Davari et al. 2019; Molino et al. 2022).

In addition to compositional changes, defatted rice bran (DRB) supplementation markedly improved the structural complexity and functional stability of the gut microbiota, as evidenced by microbial co‐occurrence network analysis. In the fecal microbial network, major hub genera like *Akkermansia*, *Lactobacillus*, *Intestinimonas*, *Streptococcus*, and uncultured species of Ruminococcaceae demonstrated a significant level of centrality in groups treated with DRB. These genera are functionally linked to mucosal healing, short‐chain fatty acid production, and immunoregulation. Their augmented network prominence signifies improved inter‐microbial collaboration and heightened ecological stability following DRB intervention (Hodgkinson et al. 2023; Tajasuwan et al. 2023; Zhu et al. 2024).

Similarly, DRB‐treated mucosal microbial networks displayed increased cohesiveness and positive connectivity. *Eubacterium ruminantium* , *Clostridium innocuum* , and *Bacteroides pectinophilus* emerged as dominant nodes, reflecting enhanced SCFA production and anti‐inflammatory potential. The predominance of intraphylum positive correlations and reduced inter‐phylum antagonism indicates a shift towards microbial mutualism, an ecological hallmark of a resilient and healthy gut ecosystem (Venegas et al. 2019).

Functionally, these network adaptations translated into significant metabolic improvements. AOM/DSS treatment resulted in the depletion of cecal SCFAs, namely, acetate, propionate, and butyrate, impairing mucosal energy supply and immune function (Li, Peng, Ding, et al. 2023; Liu et al. 2022). DRB supplementation, particularly at 6 g/kg BW, restored SCFA concentrations in a dose‐dependent manner, approaching those observed in healthy controls. Principal component analysis (PCA) of SCFA profiles further confirmed partial metabolic restoration in DRB‐treated rats, with DRB6 clustering closer to the control group.

Correlation analyses further validated the functional relevance of microbial shifts. SCFA‐producing taxa, including *Butyricicoccus*, *Ruminococcaceae_UCG‐010*, and *Coriobacteriaceae_UCG‐002*, showed strong positive correlations with host antioxidant biomarkers (GSH, SOD, CAT) and inverse correlations with oxidative stress markers (protein carbonyls, 8‐OHdG) (Zhou et al. 2023; González‐Bosch et al. 2021; Jahns et al. 2015). In contrast, proinflammatory and dysbiosis‐associated genera such as *Enterococcus* and *Caldicoprobacter* correlated negatively with antioxidant enzymes and positively with oxidative stress indicators (Huycke et al. 2002; Semenova et al. 2024).

Notably, DRB‐enriched genera such as *Streptomyces* and *Methylobacterium* demonstrated robust positive correlations with CAT activity, suggesting their involvement in microbial‐mediated redox regulation (Donald et al. 2022). At broader taxonomic levels, phyla Actinobacteriota and Bacteroidota were inversely associated with oxidative damage markers, supporting their putative role in maintaining oxidative balance (Liu et al. 2022; Semenova et al. 2024).

Together, these correlation patterns reinforce a functional microbiota–host axis, highlighting the critical role of specific microbial communities in regulating intestinal oxidative balance through modulation of SCFA production and antioxidant‐enzyme activity (González‐Bosch et al. 2021; Liu et al. 2022; Zhou et al. 2023). These insights deepen our mechanistic understanding of how DRB supplementation beneficially reshapes gut microbiota composition and metabolic outputs, ultimately supporting redox homeostasis and potentially reducing the risk of colorectal cancer (Brown et al. 2017; Tajasuwan et al. 2023).

Notwithstanding these encouraging outcomes, limitations must be recognized. Our results, predominantly obtained from rodent models, require confirmation through human clinical trials. Moreover, the specific molecular connections among DRB‐derived chemicals, gut microbiota, and host antioxidant defense systems necessitate further examination. The cumulative evidence strongly suggests that DRB possesses considerable chemopreventive potential, facilitated through antioxidative, anti‐inflammatory, and gut microbiota‐modulating mechanisms (Bardelčíková et al. 2023; Liu et al. 2022; Zhou et al. 2023). DRB's advantageous influence on SCFA synthesis, antioxidant‐enzyme activity, and gut barrier integrity underscores its potential as a dietary intervention for the prevention and management of colorectal cancer. Future research must prioritize clinical validation and comprehensive mechanistic elucidation, namely investigating the dynamics of human gut microbiota and redox homeostasis.

## 5 Conclusion

The conclusion of this study provides compelling evidence that defatted rice bran (DRB), a nutrient‐rich by‐product of rice bran oil extraction, exhibits multifunctional properties that collectively mitigate colorectal carcinogenesis in an AOM/DSS‐induced rat model. Through a combined composition of insoluble and soluble dietary fiber, phenolic compounds, flavonoids, and phytic acid, DRB delivers sustained antioxidant activity (DPPH, FRAP, and ORAC) that remains bioaccessible, mainly following gastrointestinal digestion. These bioactive components reduced intracellular ROS and modulated inflammatory responses in intestinal epithelial cells.

In vivo, a high DRB dose (6 g/kg BW/day) suppressed AOM/DSS‐induced protein and DNA oxidation and restored glutathione stores together with the SOD CAT enzymatic axis. Concomitantly, DRB reshaped gut ecology by curtailing dysbiotic Proteobacteria and enriching health‐promoting taxa such as *Akkermansia*, *Lactobacillus*, and members of the Ruminococcaceae. Network‐centrality analysis revealed tighter, predominantly positive microbial interactions on these taxa, and correlation mapping linked their expansion to heightened SCFA production and antioxidant‐enzyme activities.

Collectively, these integrated antioxidative, anti‐inflammatory, microbiota‐modulating, and network‐based mechanisms underscore DRB's potential as a multifunctional dietary component for preventive nutritional strategies targeting colorectal cancer and related inflammatory disorders. Future research should prioritize clinical validation, detailed molecular mechanistic studies, and practical applications to effectively integrate DRB into dietary interventions.

## Author Contributions

**Kansuda Wunjuntuk:** conceptualization (equal), data curation (equal), investigation (equal), methodology (equal), resources (equal), validation (equal), visualization (lead), writing – original draft (lead). **Laleewan Tajasuwan:** conceptualization (equal), data curation (equal), investigation (lead), methodology (equal), visualization (equal), writing – review and editing (equal). **Aikkarach Kettawan:** conceptualization (equal), funding acquisition (lead), investigation (equal), resources (equal), supervision (lead), writing – review and editing (lead). **Thanaporn Rungruang:** conceptualization (equal), resources (equal), supervision (equal). **Pinidphon Prombutara:** data curation (equal), investigation (equal), methodology (equal). **Pattaneeya Prangthip:** methodology (equal), validation (equal). **Akkarapol Chaisri:** methodology (equal). **Nilesh Nirmal:** validation (equal), visualization (equal), writing – review and editing (equal). **Aurawan Kringkasemsee Kettawan:** methodology (equal).

## Conflicts of Interest

The authors declare no conflicts of interest. Thai Ruam Jai Vegetable Oil Co. Ltd. supplied the defatted rice bran used in this study but had no role in the design, execution, interpretation, or reporting of the research.

## Supporting information


Table S1.–S6.

## Data Availability

The datasets generated for the current study are available from the corresponding author upon reasonable request.
