# Supplementary material for: Antioxidative Effects of Defatted Rice Bran in Rats on AOM/DSS‐Induced Colon Oxidative Stress and Correlations Between Gut Microbiota and Antioxidant Biomarkers
Source: Food Sci Nutr. 2025 Jul 29;13(8):e70554. doi: 10.1002/fsn3.70554 (PMC12305462; doi:10.1002/fsn3.70554)
Supplement: Supplementary file 1 — Table S1.–S6. [file FSN3-13-e70554-s001.docx]

# **Table S1**. Sample Size Calculation Summary

| **List** | **Details** |
| --- | --- |
| Model: | AOM/DSS‑induced colorectal cancer in rats |
| Primary endpoint: | Protein carbonyls (nmol/mg protein) |
| Reference Study | Cid-Gallegos, M. S., Sánchez-Chino, X. M., Álvarez-González, I., Madrigal-Bujaidar, E., Vásquez-Garzón, V. R., Baltiérrez-Hoyos, R., Villa-Treviño, S., Dávila-Ortíz, G., & Jiménez-Martínez, C. (2020). Modification of in vitro and in vivo antioxidant activity by consumption of cooked chickpea in a colon cancer model. Nutrients, 12(9), 2572. https://doi.org/10.3390/nu12092572 |
| Power Analysis Method |  |
| Test: | Two-sample t-test (independent groups, two-sided) |
| Software: | G*Power 3.1 |
| Effect size: | Cohen’s d = (Mean_control - Mean_treatment) / Pooled SD  = (10.5 - 7.4) / 2.10 = 1.48 |
| Using G*Power 3.1 (two-tailed t-test, α = 0.05, power = 0.80), the minimum sample size per group required was 5 rats/group.  This sample size provides adequate statistical power and is consistent with ethical research practice (3Rs principle). | |

**Table S2.** Relative Abundance at Phylum Level

| **Taxa** | | **Control** | **AOM/DSS** | | **AOM/DSS+DRB3** | | **AOM/DSS+DRB6** | |
| --- | --- | --- | --- | --- | --- | --- | --- | --- |
| **FECAL SAMPLE** |  | | |  | |  | |  |
| Firmicutes | | 58.48 | 63.08 | | 68.66 | | 63.66 | |
| Verrucomicrobia | | 23.02 | 20.25 | | 16.29 | | 19.87 | |
| Bacteroidetes | | 16.02 | 14.58 | | 13.06 | | 14.40 | |
| Actinobacteria | | 1.84 | 1.43 | | 1.54 | | 1.46 | |
| Proteobacteria | | 0.48 | 0.52 | | 0.28 | | 0.44 | |
| Patescibacteria | | 0.15 | 0.13 | | 0.17 | | 0.17 | |
| Cyanobacteria | | 0.00 | 0.00 | | 0.00 | | 0.00 | |
| Fibrobacteres | | 0.00 | 0.00 | | 0.00 | | 0.00 | |
| Fusobacteria | | 0.00 | 0.00 | | 0.00 | | 0.00 | |
|  | |  |  | |  | |  | |
| **MUCOSAL SAMPLE** |  | | |  | |  | |  |
| Firmicutes | | 55.45 | 54.50 | | 56.88 | | 55.62 | |
| Verrucomicrobia | | 11.32 | 11.05 | | 9.03 | | 12.86 | |
| Bacteroidetes | | 24.64 | 17.74 | | 22.60 | | 22.96 | |
| Actinobacteria | | 2.99 | 1.17 | | 1.98 | | 1.97 | |
| Proteobacteria | | 5.49 | 15.48 | | 9.36 | | 6.50 | |
| Patescibacteria | | 0.06 | 0.06 | | 0.13 | | 0.07 | |
| Cyanobacteria | | 0.00 | 0.00 | | 0.00 | | 0.02 | |
| Fibrobacteres | | 0.02 | 0.00 | | 0.00 | | 0.00 | |
| Fusobacteria | | 0.02 | 0.00 | | 0.01 | | 0.00 | |

**Note:** Supplementary Tables S2 present the detailed relative abundance of gut microbiota at the phylum levels corresponding to the circular plots in Figure 5.

**Table S3.** Relative Abundance at Family Level

| **Taxa** | **Control** | **AOM/DSS** | **AOM/DSS+DRB3** | **AOM/DSS+DRB6** |
| --- | --- | --- | --- | --- |
| **FECAL SAMPLE** |  |  |  |  |
| ;_[c_Bacilli] | 0.30 | 0.21 | 0.23 | 0.19 |
| Bacillaceae | 0.00 | 0.02 | 0.02 | 0.00 |
| Erysipelatoclostridiaceae | 0.04 | 0.04 | 0.04 | 0.03 |
| Erysipelotrichaceae | 4.10 | 5.02 | 4.26 | 3.35 |
| Enterococcaceae | 0.04 | 0.29 | 0.18 | 0.26 |
| Lactobacillaceae | 10.05 | 6.99 | 9.09 | 8.86 |
| Streptococcaceae | 0.00 | 0.01 | 0.01 | 0.01 |
| RF39 | 0.00 | 0.01 | 0.00 | 0.00 |
| Staphylococcaceae | 0.04 | 0.02 | 0.02 | 0.04 |
| ;_[c_Clostridia] | 0.01 | 0.05 | 0.02 | 0.02 |
| Caldicoprobacteraceae | 0.01 | 0.01 | 0.00 | 0.00 |
| Christensenellaceae | 0.13 | 0.09 | 0.09 | 0.12 |
| Clostridia_vadinBB60_group | 0.10 | 0.21 | 0.18 | 0.18 |
| Clostridiaceae | 3.60 | 5.11 | 4.13 | 3.18 |
| Anaerofustaceae | 0.00 | 0.00 | 0.00 | 0.00 |
| Defluviitaleaceae | 0.01 | 0.03 | 0.03 | 0.02 |
| Lachnospiraceae | 18.46 | 22.70 | 27.31 | 25.06 |
| Eubacteriaceae | 1.86 | 0.00 | 0.00 | 0.00 |
| Monoglobaceae | 0.01 | 0.01 | 0.01 | 0.00 |
| Butyricicoccaceae | 0.02 | 0.02 | 0.03 | 0.03 |
| Oscillospiraceae | 2.92 | 4.48 | 5.11 | 4.66 |
| Ruminococcaceae | 4.44 | 2.99 | 3.87 | 3.81 |
| Peptococcaceae | 0.71 | 0.81 | 0.77 | 0.83 |
| Anaerovoracaceae | 0.73 | 0.69 | 0.78 | 0.62 |
| Peptostreptococcaceae | 10.66 | 12.69 | 12.10 | 11.94 |
| DTU014 | 0.23 | 0.16 | 0.14 | 0.13 |
| Akkermansiaceae | 23.02 | 20.25 | 16.29 | 19.87 |
| Bacteroidaceae | 1.68 | 1.81 | 1.37 | 1.59 |
| Muribaculaceae | 10.23 | 8.94 | 7.41 | 7.70 |
| Prevotellaceae | 3.04 | 3.21 | 3.71 | 4.67 |
| Rikenellaceae | 1.07 | 1.05 | 0.78 | 0.78 |
| Corynebacteriaceae | 0.01 | 0.01 | 0.03 | 0.03 |
| Mycobacteriaceae | 0.00 | 0.00 | 0.00 | 0.00 |
| Atopobiaceae | 0.17 | 0.13 | 0.06 | 0.04 |
| Eggerthellaceae | 1.66 | 1.29 | 1.45 | 1.38 |
| uncultured[p_Proteobacteria] | 0.02 | 0.02 | 0.02 | 0.02 |
| Anaplasmataceae | 0.00 | 0.00 | 0.00 | 0.00 |
| Sutterellaceae | 0.42 | 0.24 | 0.19 | 0.25 |
| Enterobacteriaceae | 0.02 | 0.25 | 0.06 | 0.16 |
| Morganellaceae | 0.00 | 0.00 | 0.00 | 0.01 |
| Pseudomonadaceae | 0.02 | 0.00 | 0.00 | 0.00 |
| Saccharimonadaceae | 0.15 | 0.13 | 0.17 | 0.17 |
|  |  |  |  |  |
| **MUCOSAL SAMPLE** |  |  |  |  |
| Lachnospiraceae | 17.97 | 17.53 | 14.07 | 17.55 |
| Ruminococcaceae | 14.67 | 12.06 | 12.49 | 14.76 |
| Lactobacillaceae | 9.33 | 5.07 | 8.47 | 7.19 |
| Peptostreptococcaceae | 6.21 | 6.96 | 7.67 | 4.69 |
| Erysipelotrichaceae | 3.54 | 2.88 | 3.36 | 2.11 |
| Clostridiaceae | 1.62 | 2.02 | 1.40 | 1.04 |
| Clostridiales_vadinBB60_group | 0.71 | 1.19 | 1.38 | 1.11 |
| Peptococcaceae | 0.49 | 0.44 | 0.43 | 0.45 |
| Family_XIII | 0.45 | 0.29 | 0.32 | 0.27 |
| Christensenellaceae | 0.18 | 0.15 | 0.10 | 0.13 |
| Enterococcaceae | 0.10 | 5.47 | 6.88 | 6.16 |
| ;_[c_Clostridia] | 0.09 | 0.07 | 0.09 | 0.03 |
| Defluviitaleaceae | 0.04 | 0.04 | 0.03 | 0.03 |
| Streptococcaceae | 0.02 | 0.00 | 0.00 | 0.01 |
| Planococcaceae | 0.02 | 0.00 | 0.00 | 0.00 |
| Acidaminococcaceae | 0.01 | 0.00 | 0.00 | 0.00 |
| Staphylococcaceae | 0.00 | 0.25 | 0.14 | 0.10 |
| Bacillaceae | 0.00 | 0.03 | 0.05 | 0.00 |
| Paenibacillaceae | 0.00 | 0.00 | 0.00 | 0.00 |
| Caldicoprobacteraceae | 0.00 | 0.03 | 0.01 | 0.01 |
| Gracilibacteraceae | 0.00 | 0.00 | 0.00 | 0.00 |
| Veillonellaceae | 0.00 | 0.00 | 0.00 | 0.00 |
| Akkermansiaceae | 11.32 | 11.05 | 9.03 | 12.86 |
| Muribaculaceae | 15.37 | 10.06 | 13.15 | 11.87 |
| Prevotellaceae | 6.71 | 3.91 | 5.85 | 6.82 |
| Bacteroidaceae | 1.59 | 2.76 | 2.52 | 3.34 |
| Rikenellaceae | 0.95 | 0.97 | 1.08 | 0.88 |
| Barnesiellaceae | 0.02 | 0.00 | 0.00 | 0.03 |
| Marinifilaceae | 0.00 | 0.02 | 0.00 | 0.01 |
| Tannerellaceae | 0.00 | 0.00 | 0.00 | 0.00 |
| Flavobacteriaceae | 0.00 | 0.02 | 0.00 | 0.01 |
| Weeksellaceae | 0.00 | 0.01 | 0.00 | 0.00 |
| Eggerthellaceae | 2.64 | 1.04 | 1.80 | 1.81 |
| Atopobiaceae | 0.20 | 0.01 | 0.11 | 0.09 |
| Nocardiaceae | 0.10 | 0.05 | 0.05 | 0.04 |
| Microbacteriaceae | 0.03 | 0.00 | 0.00 | 0.00 |
| Streptomycetaceae | 0.02 | 0.03 | 0.00 | 0.01 |
| Corynebacteriaceae | 0.00 | 0.02 | 0.02 | 0.02 |
| Mycobacteriaceae | 0.00 | 0.00 | 0.00 | 0.00 |
| Micromonosporaceae | 0.00 | 0.01 | 0.00 | 0.01 |
| Caulobacteraceae | 2.94 | 1.84 | 0.19 | 0.27 |
| Burkholderiaceae | 1.46 | 0.47 | 0.39 | 0.59 |
| Pseudomonadaceae | 0.53 | 0.18 | 0.08 | 0.10 |
| Beijerinckiaceae | 0.38 | 0.13 | 0.02 | 0.03 |
| Enterobacteriaceae | 0.09 | 12.76 | 8.59 | 5.39 |
| Sphingomonadaceae | 0.05 | 0.01 | 0.00 | 0.00 |
| ;_[c_ Alphaproteobacteria] | 0.04 | 0.07 | 0.09 | 0.12 |
| Moraxellaceae | 0.00 | 0.02 | 0.01 | 0.00 |
| Desulfovibrionaceae | 0.00 | 0.00 | 0.00 | 0.00 |
| Aeromonadaceae | 0.00 | 0.00 | 0.00 | 0.00 |
| Saccharimonadaceae | 0.06 | 0.06 | 0.13 | 0.07 |
| Limnotrichaceae | 0.00 | 0.00 | 0.00 | 0.00 |
| Cyanobiaceae | 0.00 | 0.00 | 0.00 | 0.02 |
| Fibrobacteraceae | 0.02 | 0.00 | 0.00 | 0.00 |
| Fusobacteriaceae | 0.02 | 0.00 | 0.01 | 0.00 |
| Anaeroplasmataceae | 0.00 | 0.00 | 0.00 | 0.00 |
| Mycoplasmataceae | 0.00 | 0.00 | 0.00 | 0.00 |

**Note:** Supplementary Tables S3 present the detailed relative abundance of gut microbiota at the family levels corresponding to the circular plots in Figure 5.

**Table S4.** Relative Abundance at Genus Level

| **Taxa** | **Control** | **AOM/DSS** | **AOM/DSS+DRB3** | **AOM/DSS+DRB6** | |
| --- | --- | --- | --- | --- | --- |
| **FECAL SAMPLE** |  |  |  |  | |
| ;_[c_Bacilli] | 0.3040 | 0.2150 | 0.2322 | 0.1871 | |
| Bacillus | 0.0035 | 0.0218 | 0.0217 | 0.0000 | |
| Candidatus_Stoquefichus | 0.0091 | 0.0038 | 0.0070 | 0.0012 | |
| Erysipelatoclostridium | 0.0310 | 0.0345 | 0.0364 | 0.0270 | |
| Dubosiella | 0.5792 | 0.9417 | 0.1578 | 0.2053 | |
| Erysipelotrichaceae | 0.0000 | 0.0000 | 0.0070 | 0.0018 | |
| Turicibacter | 3.4982 | 4.0605 | 4.0775 | 3.1158 | |
| uncultured | 0.0232 | 0.0166 | 0.0129 | 0.0317 | |
| Enterococcus | 0.0443 | 0.2911 | 0.1824 | 0.2639 | |
| Lactobacillus | 10.0535 | 6.9861 | 9.0922 | 8.8576 | |
| Streptococcus | 0.0028 | 0.0051 | 0.0053 | 0.0088 | |
| RF39 | 0.0042 | 0.0051 | 0.0000 | 0.0000 | |
| Jeotgalicoccus | 0.0007 | 0.0013 | 0.0070 | 0.0106 | |
| Staphylococcus | 0.0359 | 0.0141 | 0.0164 | 0.0264 | |
| ;__[c_Clostridia] | 0.0141 | 0.0467 | 0.0158 | 0.0188 | |
| Caldicoprobacter | 0.0056 | 0.0134 | 0.0000 | 0.0006 | |
| Christensenellaceae_R-7 | 0.1105 | 0.0742 | 0.0651 | 0.0915 | |
| uncultured[c_Clostridia] | 0.0218 | 0.0205 | 0.0281 | 0.0258 | |
| Clostridia_vadinBB60 | 0.0978 | 0.2118 | 0.1812 | 0.1783 | |
| Clostridium_sensu_stricto_1 | 2.4581 | 4.0164 | 3.1457 | 2.1851 | |
| Clostridia_UCG-010 | 0.4778 | 0.4485 | 0.3407 | 0.2404 | |
| Clostridia_UCG-014 | 0.6608 | 0.6430 | 0.6480 | 0.7559 | |
| Anaerofustis | 0.0014 | 0.0032 | 0.0023 | 0.0000 | |
| Defluviitaleaceae_UCG-011 | 0.0091 | 0.0313 | 0.0264 | 0.0170 | |
| ;__[f_Lachnospiraceae] | 9.9029 | 10.3947 | 11.0099 | 10.0932 | |
| A2 | 0.2519 | 0.2373 | 0.2938 | 0.3067 | |
| ASF356 | 0.2913 | 0.2246 | 0.3085 | 0.2357 | |
| Acetatifactor | 0.0830 | 0.1657 | 0.2064 | 0.1836 | |
| Blautia | 0.3955 | 0.8445 | 0.6633 | 0.7653 | |
| GCA-900066575 | 0.3997 | 0.5572 | 0.5114 | 0.5970 | |
| Lachnoclostridium | 0.4645 | 0.4945 | 0.5847 | 0.4873 | |
| Lachnospiraceae_FCS020_group | 0.0788 | 0.1190 | 0.1085 | 0.1003 | |
| Lachnospiraceae_NK4A136_group | 3.0148 | 3.9473 | 6.4497 | 5.9102 | |
| Lachnospiraceae_NK4B4_group | 0.1661 | 0.2578 | 0.5125 | 0.6163 | |
| Lachnospiraceae_UCG-001 | 0.0176 | 0.0704 | 0.0692 | 0.0299 | |
| Lachnospiraceae_UCG-006 | 1.2632 | 0.7524 | 0.9172 | 0.7295 | |
| Marvinbryantia | 0.0823 | 0.0710 | 0.0827 | 0.0534 | |
| Roseburia | 0.0134 | 0.0550 | 0.0434 | 0.0528 | |
| Tuzzerella | 0.0816 | 0.0569 | 0.1126 | 0.1214 | |
| [Eubacterium]_coprostanoligenes_group | 1.9268 | 1.9212 | 2.2865 | 2.2953 | |
| [Eubacterium]_siraeum_group | 0.0127 | 0.0166 | 0.0147 | 0.0129 | |
| Negativibacillus | 0.0007 | 0.0051 | 0.0018 | 0.0018 | |
| Uncultured[f_Lachnospiraceae] | 0.0169 | 0.0621 | 0.0358 | 0.0387 | |
| [Eubacterium]_ruminantium_group | 1.3878 | 1.5424 | 1.9224 | 1.5148 | |
| [Eubacterium]_xylanophilum_group | 0.4694 | 0.9046 | 1.1787 | 0.9172 | |
| Monoglobus | 0.0063 | 0.0090 | 0.0065 | 0.0047 | |
| Butyricicoccus | 0.0183 | 0.0186 | 0.0334 | 0.0281 | |
| ;__[f_Oscillospiraceae] | 1.3603 | 1.9992 | 1.9358 | 1.9593 | |
| Anaerotruncus | 0.0274 | 0.0768 | 0.0833 | 0.0463 | |
| Colidextribacter | 0.4588 | 1.1720 | 1.4139 | 1.2960 | |
| Intestinimonas | 0.2864 | 0.2290 | 0.1865 | 0.1736 | |
| NK4A214_group | 0.2393 | 0.2047 | 0.2551 | 0.2182 | |
| Oscillibacter | 0.2245 | 0.3288 | 0.5102 | 0.3571 | |
| uncultured[f_Oscillospiraceae] | 0.3216 | 0.4651 | 0.7278 | 0.6070 | |
| ;__[f_Ruminococcaceae] | 2.7326 | 1.5271 | 1.8919 | 1.7013 | |
| Candidatus_Soleaferrea | 0.0373 | 0.0211 | 0.0152 | 0.0088 | |
| Harryflintia | 0.0626 | 0.0704 | 0.0721 | 0.0598 | |
| Incertae_Sedis | 0.3125 | 0.3103 | 0.3478 | 0.3331 | |
| Paludicola | 0.1070 | 0.1164 | 0.1155 | 0.0715 | |
| Pygmaiobacter | 0.0408 | 0.0608 | 0.0680 | 0.0663 | |
| Ruminococcaceae | 0.0338 | 0.0198 | 0.0751 | 0.1419 | |
| Ruminococcus | 0.8135 | 0.5233 | 0.8245 | 0.9829 | |
| [Ruminococcus]_torques_group | 0.0021 | 0.0013 | 0.0023 | 0.0000 | |
| UBA1819 | 0.0408 | 0.0320 | 0.0299 | 0.0229 | |
| uncultured[f_Ruminococcaceae] | 0.2041 | 0.1823 | 0.2258 | 0.2094 | |
| UCG-009 | 0.0141 | 0.0512 | 0.0997 | 0.0897 | |
| UCG-003 | 0.0225 | 0.0537 | 0.0886 | 0.1079 | |
| UCG-005 | 0.0155 | 0.0230 | 0.0164 | 0.0164 | |
| Peptococcus | 0.0099 | 0.0064 | 0.0123 | 0.0100 | |
| uncultured[f_Peptococcaceae] | 0.6995 | 0.8048 | 0.7571 | 0.8169 | |
| Family_XIII_AD3011_group | 0.0908 | 0.0774 | 0.0715 | 0.0569 | |
| Family_XIII_UCG-001 | 0.0345 | 0.0307 | 0.0405 | 0.0399 | |
| [Eubacterium]_brachy_group | 0.1112 | 0.0953 | 0.1372 | 0.0710 | |
| [Eubacterium]_nodatum_group | 0.4982 | 0.4888 | 0.5266 | 0.4527 | |
| Romboutsia | 10.6559 | 12.6934 | 12.1018 | 11.9376 | |
| DTU014 | 0.2315 | 0.1567 | 0.1443 | 0.1267 | |
| Akkermansia | 23.0190 | 20.2540 | 16.2949 | 19.8727 | |
| Bacteroides | 1.4068 | 1.3819 | 1.1518 | 1.2550 | |
| [Bacteroides]_pectinophilus_group | 0.2738 | 0.4280 | 0.2223 | 0.3325 | |
| ;__[f_Muribaculaceae] | 0.1372 | 0.1299 | 0.0581 | 0.1443 | |
| Muribaculaceae | 9.2780 | 8.0974 | 6.7775 | 6.9658 | |
| Muribaculum | 0.8177 | 0.7082 | 0.5771 | 0.5923 | |
| Alloprevotella | 2.4018 | 2.6006 | 2.7791 | 3.0454 | |
| Prevotellaceae_UCG-001 | 0.6362 | 0.6103 | 0.9283 | 1.6239 | |
| Alistipes | 0.7361 | 0.8349 | 0.5888 | 0.5495 | |
| Rikenella | 0.3350 | 0.2156 | 0.1947 | 0.2287 | |
| Corynebacterium | 0.0148 | 0.0070 | 0.0287 | 0.0328 | |
| Mycobacterium | 0.0021 | 0.0032 | 0.0023 | 0.0012 | |
| Coriobacteriaceae_UCG-002 | 0.1668 | 0.1337 | 0.0557 | 0.0416 | |
| Adlercreutzia | 0.0190 | 0.0000 | 0.0012 | 0.0000 | |
| Enterorhabdus | 1.3716 | 1.0857 | 1.2380 | 1.2221 | |
| Gordonibacter | 0.1795 | 0.1670 | 0.1607 | 0.1237 | |
| uncultured[f_Eggerthellaceae] | 0.0908 | 0.0326 | 0.0551 | 0.0340 | |
| uncultured[p_Proteobacteria] | 0.0190 | 0.0237 | 0.0176 | 0.0194 | |
| Anaplasma | 0.0042 | 0.0000 | 0.0012 | 0.0006 | |
| Parasutterella | 0.4201 | 0.2444 | 0.1935 | 0.2487 | |
| Citrobacter | 0.0000 | 0.0141 | 0.0070 | 0.0088 | |
| Escherichia-Shigella | 0.0211 | 0.2380 | 0.0557 | 0.1484 | |
| Proteus | 0.0000 | 0.0045 | 0.0012 | 0.0106 | |
| Pseudomonas | 0.0169 | 0.0000 | 0.0000 | 0.0000 | |
| Candidatus_Saccharimonas | 0.1513 | 0.1299 | 0.1701 | 0.1707 | |
|  |  |  |  |  | |
| **MUCOSAL SAMPLES** |  |  |  |  | |
| Lachnospiraceae_NK4A136_group | 5.1765 | 4.9849 | 4.4774 | 6.5718 | |
| ;__[f_Lachnospiraceae] | 3.1509 | 3.1756 | 2.6294 | 2.5136 | |
| Uncultured[f_Lachnospiraceae] | 1.8623 | 1.6604 | 1.3423 | 1.8406 | |
| Lachnoclostridium | 1.4210 | 1.4434 | 1.0389 | 0.9837 | |
| [Eubacterium]_ruminantium_group | 1.0768 | 0.7465 | 0.8311 | 0.6068 | |
| Lachnospiraceae_UCG-006 | 1.0040 | 0.5553 | 0.8164 | 0.7447 | |
| Blautia | 0.6929 | 1.0555 | 0.4450 | 0.3751 | |
| Marvinbryantia | 0.6553 | 0.2997 | 0.2795 | 0.2501 | |
| [Eubacterium]_xylanophilum_group | 0.4192 | 0.6344 | 0.5259 | 0.6142 | |
| GCA-900066575 | 0.3553 | 0.4358 | 0.1839 | 0.5645 | |
| Butyrivibrio | 0.3530 | 0.3126 | 0.1894 | 0.5075 | |
| Acetatifactor | 0.2891 | 0.3255 | 0.1802 | 0.2740 | |
| Lachnospiraceae_NK4B4_group | 0.2604 | 0.2703 | 0.1728 | 0.3696 | |
| Roseburia | 0.2317 | 0.5571 | 0.1710 | 0.5057 | |
| [Bacteroides]_pectinophilus_group | 0.1765 | 0.3622 | 0.1103 | 0.1085 | |
| Lachnospiraceae_UCG-001 | 0.1699 | 0.1894 | 0.2372 | 0.0699 | |
| Lachnospiraceae_FCS020_group | 0.1214 | 0.0552 | 0.0699 | 0.0644 | |
| [Ruminococcus]_torques_group | 0.1192 | 0.0000 | 0.0018 | 0.0827 | |
| Tyzzerella | 0.1103 | 0.0680 | 0.0533 | 0.0570 | |
| ASF356 | 0.1081 | 0.1894 | 0.1250 | 0.1158 | |
| A2 | 0.0816 | 0.0772 | 0.0736 | 0.0680 | |
| Lachnospiraceae_UCG-010 | 0.0397 | 0.0000 | 0.0000 | 0.0000 | |
| Eisenbergiella | 0.0331 | 0.0515 | 0.0055 | 0.0552 | |
| Acetitomaculum | 0.0265 | 0.0680 | 0.0662 | 0.1122 | |
| Lachnospiraceae_NC2004_group | 0.0199 | 0.0000 | 0.0000 | 0.0165 | |
| Lachnospira | 0.0154 | 0.0165 | 0.0441 | 0.0478 | |
| Lachnospiraceae_UCG-004 | 0.0000 | 0.0000 | 0.0000 | 0.0000 | |
| Lachnospiraceae_UCG-008 | 0.0000 | 0.0000 | 0.0000 | 0.0000 | |
| Sellimonas | 0.0000 | 0.0000 | 0.0000 | 0.0000 | |
| [Eubacterium]_hallii_group | 0.0000 | 0.0000 | 0.0000 | 0.0257 | |
| Ruminococcus_2 | 2.5816 | 1.0481 | 1.9344 | 0.7925 | |
| Ruminiclostridium_9 | 2.0697 | 2.2727 | 1.6696 | 1.9693 | |
| ;__[f_Ruminococcaceae] | 1.8667 | 1.8535 | 1.7744 | 2.0888 | |
| [Eubacterium]_coprostanoligenes | 1.4387 | 1.1400 | 1.3276 | 1.5740 | |
| Ruminiclostridium | 1.0966 | 1.1143 | 0.9396 | 0.9562 | |
| Oscillibacter | 0.8892 | 1.1143 | 1.2265 | 1.2871 | |
| Intestinimonas | 0.5274 | 0.5075 | 0.3714 | 0.3108 | |
| Ruminococcaceae_UCG-014 | 0.4832 | 0.3108 | 0.4303 | 0.4045 | |
| Ruminococcaceae_UCG-010 | 0.4788 | 0.3052 | 0.1655 | 0.1802 | |
| Ruminococcus_1 | 0.3729 | 0.3622 | 0.5020 | 0.6877 | |
| uncultured[f_Ruminococcaceae] | 0.3641 | 0.3457 | 0.4027 | 0.4266 | |
| Ruminococcaceae_NK4A214 | 0.3332 | 0.1508 | 0.1839 | 0.1269 | |
| Faecalibacterium | 0.3067 | 0.0552 | 0.0441 | 0.0000 | |
| Ruminiclostridium_5 | 0.3023 | 0.2519 | 0.1820 | 0.2887 | |
| GCA-900066225 | 0.2273 | 0.0901 | 0.0919 | 0.0791 | |
| Ruminococcaceae_UCG-004 | 0.2251 | 0.2188 | 0.3604 | 0.6822 | |
| Butyricicoccus | 0.1589 | 0.0772 | 0.1177 | 0.1232 | |
| Pygmaiobacter | 0.1567 | 0.0386 | 0.0496 | 0.1048 | |
| Angelakisella | 0.1280 | 0.1508 | 0.0864 | 0.0901 | |
| Ruminococcaceae_UCG-009 | 0.1236 | 0.1158 | 0.1195 | 0.0533 | |
| Ruminococcaceae_UCG-003 | 0.1037 | 0.1802 | 0.2059 | 0.2446 | |
| Ruminococcaceae_UCG-005 | 0.0838 | 0.0772 | 0.0331 | 0.0074 | |
| Harryflintia | 0.0662 | 0.0772 | 0.0901 | 0.0588 | |
| Subdoligranulum | 0.0596 | 0.0110 | 0.0000 | 0.0000 | |
| Ruminiclostridium_6 | 0.0574 | 0.0129 | 0.0423 | 0.0717 | |
| Ruminococcaceae_UCG-002 | 0.0508 | 0.0000 | 0.0000 | 0.0000 | |
| Negativibacillus | 0.0441 | 0.0092 | 0.0000 | 0.0000 | |
| UBA1819 | 0.0309 | 0.0846 | 0.0478 | 1.9160 | |
| Candidatus_Soleaferrea | 0.0287 | 0.0018 | 0.0092 | 0.0221 | |
| Ruminococcaceae_UCG-013 | 0.0066 | 0.0018 | 0.0129 | 0.0000 | |
| Anaerotruncus | 0.0044 | 0.0809 | 0.0699 | 0.0478 | |
| Acetanaerobacterium | 0.0000 | 0.0000 | 0.0000 | 0.0000 | |
| DTU089 | 0.0000 | 0.0000 | 0.0000 | 0.0000 | |
| Flavonifractor | 0.0000 | 0.0000 | 0.0000 | 0.1618 | |
| Fournierella | 0.0000 | 0.0000 | 0.0000 | 0.0000 | |
| Lactobacillus | 9.3270 | 5.0658 | 8.4712 | 7.1859 | |
| Romboutsia | 6.2092 | 6.9598 | 7.6732 | 4.6944 | |
| Turicibacter | 2.8045 | 2.6662 | 3.2436 | 1.9050 | |
| Dubosiella | 0.6730 | 0.1673 | 0.0754 | 0.1710 | |
| Erysipelatoclostridium | 0.0331 | 0.0368 | 0.0129 | 0.0276 | |
| uncultured[f_Erysipelotrichaceae] | 0.0199 | 0.0055 | 0.0221 | 0.0000 | |
| Candidatus_Stoquefichus | 0.0066 | 0.0018 | 0.0018 | 0.0000 | |
| Holdemania | 0.0044 | 0.0000 | 0.0037 | 0.0000 | |
| [Clostridium]_innocuum_group | 0.0000 | 0.0000 | 0.0000 | 0.0037 | |
| Clostridium_sensu_stricto_1 | 1.5622 | 2.0006 | 1.3993 | 1.0389 | |
| Candidatus_Arthromitus | 0.0574 | 0.0202 | 0.0000 | 0.0037 | |
| Ambiguous_taxa[f_Clostridiales_vadinBB60] | 0.3398 | 0.8127 | 0.8238 | 0.6344 | |
| Uncultured[f_Clostridiales_vadinBB60] | 0.2714 | 0.2832 | 0.4248 | 0.3622 | |
| ;__[f_Clostridiales_vadinBB60] | 0.0640 | 0.0846 | 0.1250 | 0.1048 | |
| gut_metagenome | 0.0243 | 0.0055 | 0.0018 | 0.0000 | |
| uncultured_Clostridia_bacterium | 0.0132 | 0.0055 | 0.0000 | 0.0074 | |
| uncultured_Firmicutes_bacterium | 0.0000 | 0.0000 | 0.0000 | 0.0000 | |
| uncultured[f_Peptococcaceae] | 0.4854 | 0.4395 | 0.4321 | 0.4450 | |
| Peptococcus | 0.0000 | 0.0037 | 0.0018 | 0.0018 | |
| [Eubacterium]_nodatum_group | 0.3023 | 0.1949 | 0.2188 | 0.1820 | |
| [Eubacterium]_brachy_group | 0.1015 | 0.0441 | 0.0533 | 0.0552 | |
| Family_XIII_UCG-001 | 0.0177 | 0.0092 | 0.0037 | 0.0110 | |
| Anaerovorax | 0.0154 | 0.0184 | 0.0202 | 0.0018 | |
| Family_XIII_AD3011_group | 0.0088 | 0.0276 | 0.0221 | 0.0221 | |
| Christensenellaceae_R-7_group | 0.1258 | 0.0644 | 0.0460 | 0.0938 | |
| ;__[f_Christensenellaceae] | 0.0353 | 0.0644 | 0.0129 | 0.0110 | |
| uncultured[f_Christensenellaceae] | 0.0199 | 0.0221 | 0.0405 | 0.0221 | |
| Enterococcus | 0.1037 | 5.4722 | 6.8825 | 6.1636 | |
| Ambiguous_taxa[c_Clostridia] | 0.0883 | 0.0699 | 0.0864 | 0.0294 | |
| Defluviitaleaceae_UCG-011 | 0.0419 | 0.0441 | 0.0313 | 0.0276 | |
| Streptococcus | 0.0243 | 0.0000 | 0.0000 | 0.0074 | |
| ;__[f_Planococcaceae] | 0.0199 | 0.0000 | 0.0000 | 0.0000 | |
| Acidaminococcus | 0.0110 | 0.0000 | 0.0000 | 0.0000 | |
| Phascolarctobacterium | 0.0000 | 0.0000 | 0.0000 | 0.0000 | |
| Staphylococcus | 0.0044 | 0.2519 | 0.1379 | 0.0993 | |
| Bacillus | 0.0000 | 0.0349 | 0.0496 | 0.0000 | |
| Paenibacillus | 0.0000 | 0.0000 | 0.0000 | 0.0000 | |
| Caldicoprobacter | 0.0000 | 0.0294 | 0.0055 | 0.0092 | |
| Gracilibacter | 0.0000 | 0.0000 | 0.0000 | 0.0000 | |
| Megasphaera | 0.0000 | 0.0000 | 0.0000 | 0.0018 | |
| Veillonella | 0.0000 | 0.0000 | 0.0000 | 0.0000 | |
| Akkermansia | 11.3239 | 11.0510 | 9.0339 | 12.8604 | |
| Ambiguous_taxa[f_Muribaculaceae] | 2.5088 | 1.7303 | 1.8369 | 1.5133 | |
| Muribaculum | 0.4987 | 0.2942 | 0.4192 | 0.2795 | |
| metagenome | 0.0309 | 0.0000 | 0.0000 | 0.0000 | |
| uncultured_Bacteroidales [f_Muribaculaceae] | 1.4784 | 1.2614 | 1.8020 | 1.5850 | |
| uncultured[f_Muribaculaceae] | 4.1196 | 3.1333 | 3.8835 | 3.6500 | |
| ;__[f_Muribaculaceae] | 6.7343 | 3.6371 | 5.2129 | 4.8433 | |
| Alloprevotella | 5.6134 | 3.2565 | 4.1924 | 4.1409 | |
| Prevotella_9 | 0.0838 | 0.0165 | 0.0074 | 0.0074 | |
| Prevotellaceae_UCG-001 | 1.0150 | 0.6344 | 1.6494 | 2.6699 | |
| Bacteroides | 1.5887 | 2.7618 | 2.5173 | 3.3392 | |
| Alistipes | 0.8230 | 0.8256 | 0.9304 | 0.7649 | |
| Rikenella | 0.1236 | 0.1416 | 0.1471 | 0.1140 | |
| ;__[f_Rikenellaceae] | 0.0000 | 0.0000 | 0.0000 | 0.0000 | |
| Coprobacter | 0.0221 | 0.0000 | 0.0000 | 0.0294 | |
| Odoribacter | 0.0000 | 0.0202 | 0.0037 | 0.0129 | |
| Parabacteroides | 0.0000 | 0.0000 | 0.0000 | 0.0000 | |
| Vitellibacter | 0.0000 | 0.0202 | 0.0000 | 0.0074 | |
| Cloacibacterium | 0.0000 | 0.0055 | 0.0000 | 0.0000 | |
| Adlercreutzia | 0.0441 | 0.0000 | 0.0129 | 0.0018 | |
| Eggerthella | 0.0000 | 0.0239 | 0.0147 | 0.4818 | |
| Enterorhabdus | 1.7851 | 0.7043 | 1.0941 | 0.7870 | |
| Gordonibacter | 0.2361 | 0.1287 | 0.2023 | 0.1508 | |
| Uncultured[f_Eggerthellaceae] | 0.1831 | 0.0441 | 0.1085 | 0.0662 |  |
| ;__[f_Eggerthellaceae] | 0.3950 | 0.1361 | 0.3659 | 0.3181 |  |
| Coriobacteriaceae_UCG-002 | 0.1964 | 0.0129 | 0.1122 | 0.0864 |  |
| Rhodococcus | 0.0971 | 0.0478 | 0.0460 | 0.0405 |  |
| ;__[f_Microbacteriaceae] | 0.0265 | 0.0000 | 0.0000 | 0.0000 |  |
| Streptomyces | 0.0243 | 0.0331 | 0.0037 | 0.0055 |  |
| Corynebacterium_1 | 0.0044 | 0.0221 | 0.0221 | 0.0239 |  |
| Mycobacterium | 0.0000 | 0.0037 | 0.0000 | 0.0000 |  |
| Planosporangium | 0.0000 | 0.0110 | 0.0018 | 0.0074 |  |
| Brevundimonas | 2.9391 | 1.8351 | 0.1931 | 0.2740 |  |
| Burkholderia-Caballeronia-Paraburkholderia | 0.1523 | 0.0699 | 0.0184 | 0.0055 |  |
| Delftia | 0.3508 | 0.1140 | 0.0699 | 0.0717 |  |
| MWH-UniP1_aquatic_group | 0.0419 | 0.0000 | 0.0000 | 0.0000 |  |
| Parasutterella | 0.7370 | 0.2611 | 0.2703 | 0.4395 |  |
| Ralstonia | 0.1765 | 0.0294 | 0.0276 | 0.0717 |  |
| Pseudomonas | 0.5318 | 0.1802 | 0.0754 | 0.0956 |  |
| Bosea | 0.1721 | 0.0717 | 0.0018 | 0.0074 |  |
| Methylobacterium | 0.2052 | 0.0552 | 0.0165 | 0.0257 |  |
| Citrobacter | 0.0132 | 4.3579 | 3.2804 | 1.2743 |  |
| Enterobacter | 0.0044 | 0.0000 | 0.0000 | 0.0202 |  |
| Escherichia-Shigella | 0.0618 | 6.5019 | 3.9534 | 3.4073 |  |
| Klebsiella | 0.0000 | 0.0000 | 0.0000 | 0.0000 |  |
| Proteus | 0.0044 | 0.0257 | 0.0441 | 0.2023 |  |
| ;__[f_Enterobacteriaceae] | 0.0044 | 1.8774 | 1.3147 | 0.4873 |  |
| Novosphingobium | 0.0530 | 0.0147 | 0.0000 | 0.0000 |  |
| Azospirillum_sp._47_25 | 0.0419 | 0.0680 | 0.0901 | 0.1158 |  |
| Psychrobacter | 0.0022 | 0.0202 | 0.0074 | 0.0000 |  |
| Bilophila | 0.0000 | 0.0000 | 0.0000 | 0.0000 |  |
| Aeromonas | 0.0000 | 0.0000 | 0.0000 | 0.0000 |  |
| Candidatus_Saccharimonas | 0.0574 | 0.0552 | 0.1250 | 0.0680 |  |
| Limnothrix | 0.0000 | 0.0000 | 0.0000 | 0.0000 |  |
| Cyanobium_PCC-6307 | 0.0000 | 0.0000 | 0.0000 | 0.0221 |  |
| uncultured[f_Fibrobacteraceae] | 0.0199 | 0.0000 | 0.0000 | 0.0000 |  |
| Cetobacterium | 0.0000 | 0.0037 | 0.0000 | 0.0000 |  |
| Fusobacterium | 0.0221 | 0.0000 | 0.0074 | 0.0000 |  |
| Anaeroplasma | 0.0000 | 0.0000 | 0.0000 | 0.0000 |  |
| Candidatus_Bacilloplasma | 0.0000 | 0.0000 | 0.0000 | 0.0000 |  |

**Note:** Supplementary Tables S4 present the detailed relative abundance of gut microbiota at the genus levels corresponding to the circular plots in Figure 5.

**Table S5**. Ranked Centrality Metrics of Bacterial Genera in Faecal Samples.

| **Genus** | **Degree Centrality** | **Betweenness Centrality** | **Closeness Centrality** | **Rank** |
| --- | --- | --- | --- | --- |
| *Akkermansia* | 0.12 | 0 | 0.12 | 20 |
| *Intestinimonas* | 0.12 | 0 | 0.12 | 20 |
| *Lactobacillus* | 0.12 | 0 | 0.12 | 20 |
| *Streptococcus* | 0.12 | 0 | 0.12 | 20 |
| ;__[f_Ruminococcaceae] | 0.12 | 0 | 0.12 | 20 |
| *Jeotgalicoccus* | 0.12 | 0 | 0.12 | 20 |
| uncultured[f_Oscillospiraceae] | 0.12 | 0 | 0.12 | 20 |
| ;__[c_Clostridia] | 0.12 | 0 | 0.12 | 20 |
| *UCG-003* | 0.12 | 0 | 0.12 | 20 |
| *Oscillibacter* | 0.12 | 0 | 0.12 | 20 |
| *Colidextribacter* | 0.12 | 0 | 0.12 | 20 |
| *Lachnospiraceae_NK4A136* | 0.12 | 0 | 0.12 | 20 |
| ;__[f_Oscillospiraceae] | 0.12 | 0 | 0.12 | 20 |
| *UCG-009* | 0.12 | 0 | 0.12 | 20 |
| *Butyricicoccus* | 0.12 | 0 | 0.12 | 20 |
| uncultured[f_Lachnospiraceae] | 0.12 | 0 | 0.12 | 20 |
| *[Eubacterium]_xylanophilum* | 0.12 | 0 | 0.12 | 20 |
| *Roseburia* | 0.12 | 0 | 0.12 | 20 |
| *Acetatifactor* | 0.12 | 0 | 0.12 | 20 |
| *Blautia* | 0.12 | 0 | 0.12 | 20 |
| *Enterococcus* | 0.12 | 0 | 0.12 | 20 |
| *Candidatus_Soleaferrea* | 0.12 | 0 | 0.12 | 20 |
| *Pygmaiobacter* | 0.12 | 0 | 0.12 | 20 |
| *Alloprevotella* | 0.12 | 0 | 0.12 | 20 |
| *Coriobacteriaceae_UCG-002* | 0.12 | 0 | 0.12 | 20 |
| *Escherichia-Shigella* | 0.12 | 0 | 0.12 | 20 |
| *Enterorhabdus* | 0.12 | 0 | 0.12 | 20 |
| *Gordonibacter* | 0.12 | 0 | 0.12 | 20 |
| uncultured[f_Eggerthellaceae] | 0.12 | 0 | 0.12 | 20 |
| *Bacteroides* | 0.12 | 0 | 0.12 | 20 |
| *Citrobacter* | 0.12 | 0 | 0.12 | 20 |
| *Muribaculaceae* | 0.12 | 0 | 0.12 | 20 |
| *Muribaculum* | 0.12 | 0 | 0.12 | 20 |
| *Anaplasma* | 0.12 | 0 | 0.12 | 20 |
| *DTU014* | 0.12 | 0 | 0.12 | 20 |
| *Family_XIII_AD3011* | 0.12 | 0 | 0.12 | 20 |
| *UCG-010* | 0.12 | 0 | 0.12 | 20 |
| *UBA1819* | 0.12 | 0 | 0.12 | 20 |
| *Lachnospiraceae_NK4B4* | 0.12 | 0 | 0.12 | 20 |
| *Ruminococcaceae* | 0.08 | 0 | 0.08 | 44 |
| *Candidatus_Saccharimonas* | 0.08 | 0 | 0.08 | 44 |
| *[Eubacterium]_coprostanoligenes* | 0.08 | 0 | 0.08 | 44 |
| *Tuzzerella* | 0.08 | 0 | 0.08 | 44 |
| *Ruminococcus* | 0.08 | 0 | 0.08 | 44 |
| *Corynebacterium* | 0.08 | 0 | 0.08 | 44 |
| *Prevotellaceae_UCG-001* | 0.08 | 0 | 0.08 | 44 |
| *Alistipes* | 0.08 | 0 | 0.08 | 44 |
| A2[f_Lachnospiraceae] | 0.08 | 0 | 0.08 | 44 |
| *Lachnospiraceae_FCS020* | 0.06 | 0 | 0.06 | 62 |
| Uncultured[f__Ruminococcaceae] | 0.06 | 0 | 0.06 | 62 |
| *Staphylococcus* | 0.06 | 0 | 0.06 | 62 |
| *Anaerotruncus* | 0.06 | 0 | 0.06 | 62 |
| *Lachnoclostridium* | 0.06 | 0 | 0.06 | 62 |
| *Paludicola* | 0.06 | 0 | 0.06 | 62 |
| *Dubosiella* | 0.06 | 0 | 0.06 | 62 |
| *[Eubacterium]_siraeum* | 0.06 | 0 | 0.06 | 62 |
| *Peptococcus* | 0.06 | 0 | 0.06 | 62 |
| *Bacillus* | 0.06 | 0 | 0.06 | 62 |
| *Christensenellaceae_R-7* | 0.06 | 0 | 0.06 | 62 |
| *Family_XIII_UCG-001* | 0.06 | 0 | 0.06 | 62 |
| *Romboutsia* | 0.06 | 0 | 0.06 | 62 |
| *Rikenella* | 0.06 | 0 | 0.06 | 62 |
| *Parasutterella* | 0.06 | 0 | 0.06 | 62 |
| *Caldicoprobacter* | 0.06 | 0 | 0.06 | 62 |
| *Incertae_Sedis* | 0.06 | 0 | 0.06 | 62 |
| ;__[f_Lachnospiraceae] | 0.06 | 0 | 0.06 | 62 |
| *clostridium_sensu_stricto_1* | 0.06 | 0 | 0.06 | 62 |
| *[eubacterium]_ruminantium* | 0.06 | 0 | 0.06 | 62 |
| uncultured[f_Christensenellaceae] | 0.06 | 0 | 0.06 | 62 |
| *Defluviitaleaceae_UCG-011* | 0.06 | 0 | 0.06 | 62 |
| *Monoglobus* | 0.06 | 0 | 0.06 | 62 |
| *Anaerofustis* | 0.06 | 0 | 0.06 | 62 |
| *Lachnospiraceae_UCG-001* | 0.06 | 0 | 0.06 | 62 |
| *Clostridia_vadinBB60* | 0.06 | 0 | 0.06 | 62 |
| *Clostridia_UCG-014* | 0.06 | 0 | 0.06 | 62 |
| *Mycobacterium* | 0.06 | 0 | 0.06 | 62 |
| *GCA-900066575* | 0.05 | 0 | 0.05 | 79 |
| Candidatus_Stoquefichus | 0.05 | 0 | 0.05 | 79 |
| Proteus | 0.05 | 0 | 0.05 | 79 |
| uncultured[f_Peptococcaceae] | 0.05 | 0 | 0.05 | 79 |
| ;__[c_Bacilli] | 0.05 | 0 | 0.05 | 79 |
| *Lachnospiraceae_UCG-006* | 0.05 | 0 | 0.05 | 79 |
| *Harryflintia* | 0.04 | 0 | 0.04 | 85 |
| uncultured[f_Erysipelotrichaceae] | 0.04 | 0 | 0.04 | 85 |
| *Turicibacter* | 0.04 | 0 | 0.04 | 85 |
| *Erysipelatoclostridium* | 0.04 | 0 | 0.04 | 85 |
| ;__[f_Muribaculaceae] | 0.04 | 0 | 0.04 | 85 |
| *ASF356* | 0.03 | 0 | 0.03 | 91 |
| uncultured[c_Alphaproteobacteria] | 0.03 | 0 | 0.03 | 91 |
| *NK4A214_group* | 0.03 | 0 | 0.03 | 91 |
| *[Eubacterium]_nodatum* | 0.03 | 0 | 0.03 | 91 |
| *[Eubacterium]_brachy* | 0.03 | 0 | 0.03 | 91 |
| *[Bacteroides]_pectinophilus* | 0.03 | 0 | 0.03 | 91 |
| *[Ruminococcus]_torques* | 0.03 | 0 | 0.03 | 91 |
| *Marvinbryantia* | 0.03 | 0 | 0.03 | 91 |
| *Negativibacillus* | 0.01 | 0 | 0.01 | 96 |
| *UCG-005* | 0.01 | 0 | 0.01 | 96 |
| *Erysipelotrichaceae* | 0 | 0 | 0 | 99 |
| *RF39* | 0 | 0 | 0 | 99 |
| *Adlercreutzia* | 0 | 0 | 0 | 99 |
| *Pseudomonas* | 0 | 0 | 0 | 99 |

This table presents the centrality metrics of bacterial genera identified from faecal samples across four experimental groups: Control, AOM/DSS, AOM/DSS+DRB3 (defatted rice bran 3 g), and AOM/DSS+DRB6 (defatted rice bran 6 g). Centrality measures include Degree Centrality, indicating the number of direct connections a genus has within the microbial network; Betweenness Centrality, reflecting the genus’s role as a bridge or hub within the network by quantifying how often it lies on the shortest paths between other genera; and Closeness Centrality, representing the efficiency with which a genus can interact with all other genera in the network. Genera are ranked in descending order based on their degree of centrality, with higher ranks indicating more central and potentially influential taxa within the gut microbial community.

**Table S6.** Ranked Centrality Metrics of Bacterial Genera in Mucosal Samples.

| **Genus** | **Degree Centrality** | **Betweenness Centrality** | **Closeness Centrality** | **Rank** |
| --- | --- | --- | --- | --- |
| *Coriobacteriaceae_UCG-002* | 0.1095890410958904 | 0 | 0.1095890410958904 | 9 |
| *Adlercreutzia* | 0.1095890410958904 | 0 | 0.1095890410958904 | 9 |
| *Lactobacillus* | 0.1095890410958904 | 0 | 0.1095890410958904 | 9 |
| *Caldicoprobacter* | 0.1095890410958904 | 0 | 0.1095890410958904 | 9 |
| *Enterorhabdus* | 0.1095890410958904 | 0 | 0.1095890410958904 | 9 |
| ;__[f_Muribaculaceae] | 0.1095890410958904 | 0 | 0.1095890410958904 | 9 |
| *Lachnospiraceae_UCG-006* | 0.1095890410958904 | 0 | 0.1095890410958904 | 9 |
| *Odoribacter* | 0.1095890410958904 | 0 | 0.1095890410958904 | 9 |
| ;__[f_ Eggerthellaceae] | 0.1095890410958904 | 0 | 0.1095890410958904 | 9 |
| *Alloprevotella* | 0.1095890410958904 | 0 | 0.1095890410958904 | 9 |
| *Planosporangium* | 0.1095890410958904 | 0 | 0.1095890410958904 | 9 |
| *Ruminococcaceae_UCG-014* | 0.1095890410958904 | 0 | 0.1095890410958904 | 9 |
| uncultured [f_ Eggerthellaceae] | 0.1095890410958904 | 0 | 0.1095890410958904 | 9 |
| *Lachnospiraceae_FCS020* | 0.1095890410958904 | 0 | 0.1095890410958904 | 9 |
| *Gordonibacter* | 0.1095890410958904 | 0 | 0.1095890410958904 | 9 |
| *[Eubacterium]_xylanophilum_group* | 0.1095890410958904 | 0 | 0.1095890410958904 | 9 |
| uncultured_bacterium[f_ Muribaculaceae] | 0.1095890410958904 | 0 | 0.1095890410958904 | 9 |
| *Oscillibacter* | 0.082191781 | 0 | 0.082191781 | 30 |
| gut_metagenome [f_ Clostridiales_vadinBB60 | 0.082191781 | 0 | 0.082191781 | 30 |
| *Ruminococcaceae_UCG-003* | 0.082191781 | 0 | 0.082191781 | 30 |
| *Rhodococcus* | 0.082191781 | 0 | 0.082191781 | 30 |
| Ambiguous_taxa [c_ Clostridia] | 0.082191781 | 0 | 0.082191781 | 30 |
| Ambiguous_taxa[f_ Muribaculaceae] | 0.082191781 | 0 | 0.082191781 | 30 |
| *Ruminococcaceae_UCG-005* | 0.082191781 | 0 | 0.082191781 | 30 |
| *Ruminococcaceae_UCG-009* | 0.082191781 | 0 | 0.082191781 | 30 |
| *Proteus* | 0.082191781 | 0 | 0.082191781 | 30 |
| *Intestinimonas* | 0.082191781 | 0 | 0.082191781 | 30 |
| *Bacteroides* | 0.082191781 | 0 | 0.082191781 | 30 |
| *UBA1819* | 0.082191781 | 0 | 0.082191781 | 30 |
| *Burkholderia-Caballeronia-Paraburkholderia* | 0.082191781 | 0 | 0.082191781 | 30 |
| *Lachnospira* | 0.082191781 | 0 | 0.082191781 | 30 |
| *[Eubacterium]_ruminantium* | 0.082191781 | 0 | 0.082191781 | 30 |
| A2 [f_ Lachnospiraceae] | 0.082191781 | 0 | 0.082191781 | 30 |
| *Acetitomaculum* | 0.082191781 | 0 | 0.082191781 | 30 |
| *GCA-900066225* | 0.082191781 | 0 | 0.082191781 | 30 |
| *Faecalibacterium* | 0.082191781 | 0 | 0.082191781 | 30 |
| *[Eubacterium]_nodatum* | 0.082191781 | 0 | 0.082191781 | 30 |
| *Azospirillum_sp._47_25* | 0.082191781 | 0 | 0.082191781 | 30 |
| *Ruminococcus_2* | 0.082191781 | 0 | 0.082191781 | 30 |
| *Marvinbryantia* | 0.082191781 | 0 | 0.082191781 | 30 |
| *Ruminococcaceae_NK4A214* | 0.082191781 | 0 | 0.082191781 | 30 |
| *Eggerthella* | 0.082191781 | 0 | 0.082191781 | 30 |
| *Muribaculum* | 0.082191781 | 0 | 0.082191781 | 30 |
| *Escherichia-Shigella* | 0.075342466 | 0 | 0.075342466 | 49 |
| *Pygmaiobacter* | 0.075342466 | 0 | 0.075342466 | 49 |
| *Parasutterella* | 0.075342466 | 0 | 0.075342466 | 49 |
| ;__[f_ Enterobacteriaceae] | 0.075342466 | 0 | 0.075342466 | 49 |
| *[Eubacterium]_brachy* | 0.075342466 | 0 | 0.075342466 | 49 |
| *Citrobacter* | 0.075342466 | 0 | 0.075342466 | 49 |
| *Candidatus_Soleaferrea* | 0.075342466 | 0 | 0.075342466 | 49 |
| *ASF356* | 0.075342466 | 0 | 0.075342466 | 49 |
| *Staphylococcus* | 0.075342466 | 0 | 0.075342466 | 49 |
| *Butyricicoccus* | 0.075342466 | 0 | 0.075342466 | 49 |
| *Anaerotruncus* | 0.075342466 | 0 | 0.075342466 | 49 |
| *[Ruminococcus]_torques* | 0.075342466 | 0 | 0.075342466 | 49 |
| *Lachnoclostridium* | 0.068493151 | 0 | 0.068493151 | 66 |
| *Defluviitaleaceae_UCG-011* | 0.068493151 | 0 | 0.068493151 | 66 |
| ;__[f_ Lachnospiraceae] | 0.068493151 | 0 | 0.068493151 | 66 |
| *Tyzzerella* | 0.068493151 | 0 | 0.068493151 | 66 |
| *Enterococcus* | 0.068493151 | 0 | 0.068493151 | 66 |
| *Ruminococcus_1* | 0.068493151 | 0 | 0.068493151 | 66 |
| *Blautia* | 0.068493151 | 0 | 0.068493151 | 66 |
| *Methylobacterium* | 0.068493151 | 0 | 0.068493151 | 66 |
| *Delftia* | 0.068493151 | 0 | 0.068493151 | 66 |
| *Bosea* | 0.068493151 | 0 | 0.068493151 | 66 |
| *Brevundimonas* | 0.068493151 | 0 | 0.068493151 | 66 |
| uncultured [f_ Ruminococcaceae] | 0.068493151 | 0 | 0.068493151 | 66 |
| *Prevotellaceae_UCG-001* | 0.068493151 | 0 | 0.068493151 | 66 |
| *Ruminococcaceae_UCG-010* | 0.068493151 | 0 | 0.068493151 | 66 |
| ;__[f_ Christensenellaceae] | 0.068493151 | 0 | 0.068493151 | 66 |
| *Candidatus_Arthromitus* | 0.068493151 | 0 | 0.068493151 | 66 |
| *Clostridium_sensu_stricto_1* | 0.068493151 | 0 | 0.068493151 | 66 |
| ;__[f_ Clostridiales_vadinBB60] | 0.068493151 | 0 | 0.068493151 | 66 |
| *Ruminococcaceae_UCG-004* | 0.068493151 | 0 | 0.068493151 | 66 |
| *Pseudomonas* | 0.068493151 | 0 | 0.068493151 | 66 |
| uncultured_bacterium [f_ Clostridiales_vadinBB60] | 0.068493151 | 0 | 0.068493151 | 66 |
| *[Bacteroides]_pectinophilus* | 0.068493151 | 0 | 0.068493151 | 66 |
| *Lachnospiraceae_NK4A136* | 0.061643836 | 0 | 0.061643836 | 82 |
| *Lachnospiraceae_UCG-001* | 0.061643836 | 0 | 0.061643836 | 82 |
| *Harryflintia* | 0.061643836 | 0 | 0.061643836 | 82 |
| *Akkermansia* | 0.061643836 | 0 | 0.061643836 | 82 |
| *Anaerovorax* | 0.061643836 | 0 | 0.061643836 | 82 |
| *Alistipes* | 0.061643836 | 0 | 0.061643836 | 82 |
| ;__[f_ Ruminococcaceae] | 0.061643836 | 0 | 0.061643836 | 82 |
| *Rikenella* | 0.061643836 | 0 | 0.061643836 | 82 |
| *Romboutsia* | 0.061643836 | 0 | 0.061643836 | 82 |
| *Butyrivibrio* | 0.061643836 | 0 | 0.061643836 | 82 |
| *Ruminiclostridium_5* | 0.054794521 | 0 | 0.054794521 | 92 |
| *Ralstonia* | 0.054794521 | 0 | 0.054794521 | 92 |
| uncultured [f_ Lachnospiraceae] | 0.054794521 | 0 | 0.054794521 | 92 |
| *Dubosiella* | 0.054794521 | 0 | 0.054794521 | 92 |
| *Christensenellaceae_R-7* | 0.054794521 | 0 | 0.054794521 | 92 |
| Ambiguous_taxa [f_ Clostridiales_vadinBB60] | 0.054794521 | 0 | 0.054794521 | 92 |
| uncultured_Clostridia_bacterium [f_ Clostridiales_vadinBB60] | 0.054794521 | 0 | 0.054794521 | 92 |
| *Family_XIII_UCG-001* | 0.054794521 | 0 | 0.054794521 | 92 |
| uncultured [f_ Peptococcaceae] | 0.054794521 | 0 | 0.054794521 | 92 |
| uncultured_Bacteroidales_bacterium | 0.047945205 | 0 | 0.047945205 | 104 |
| uncultured[f_ Fibrobacteraceae] | 0.047945205 | 0 | 0.047945205 | 104 |
| *Streptomyces* | 0.047945205 | 0 | 0.047945205 | 104 |
| *Ruminococcaceae_UCG-002* | 0.047945205 | 0 | 0.047945205 | 104 |
| *Ruminiclostridium_9* | 0.047945205 | 0 | 0.047945205 | 104 |
| *Ruminiclostridium* | 0.047945205 | 0 | 0.047945205 | 104 |
| *Angelakisella* | 0.047945205 | 0 | 0.047945205 | 104 |
| ;__[f_ Microbacteriaceae] | 0.047945205 | 0 | 0.047945205 | 104 |
| ;__[f_ Planococcaceae] | 0.047945205 | 0 | 0.047945205 | 104 |
| *Acidaminococcus* | 0.047945205 | 0 | 0.047945205 | 104 |
| *Candidatus_Saccharimonas* | 0.047945205 | 0 | 0.047945205 | 104 |
| *metagenome* | 0.047945205 | 0 | 0.047945205 | 104 |
| *Erysipelatoclostridium* | 0.047945205 | 0 | 0.047945205 | 104 |
| *Lachnospiraceae_UCG-010* | 0.047945205 | 0 | 0.047945205 | 104 |
| *MWH-UniP1_aquatic* | 0.047945205 | 0 | 0.047945205 | 104 |
| *Acetatifactor* | 0.047945205 | 0 | 0.047945205 | 104 |
| *Ruminococcaceae_UCG-013* | 0.034246575 | 0 | 0.034246575 | 115 |
| *Lachnospiraceae_NK4B4* | 0.034246575 | 0 | 0.034246575 | 115 |
| *Eisenbergiella* | 0.034246575 | 0 | 0.034246575 | 115 |
| *Turicibacter* | 0.034246575 | 0 | 0.034246575 | 115 |
| *GCA-900066575* | 0.034246575 | 0 | 0.034246575 | 115 |
| uncultured[f_ Erysipelotrichaceae] | 0.034246575 | 0 | 0.034246575 | 115 |
| *[Clostridium]_innocuum* | 0.02739726 | 0 | 0.02739726 | 121 |
| *Megasphaera* | 0.02739726 | 0 | 0.02739726 | 121 |
| *Cyanobium_PCC-6307* | 0.02739726 | 0 | 0.02739726 | 121 |
| *Flavonifractor* | 0.02739726 | 0 | 0.02739726 | 121 |
| *[Eubacterium]_hallii* | 0.02739726 | 0 | 0.02739726 | 121 |
| *Subdoligranulum* | 0.020547945 | 0 | 0.020547945 | 125 |
| *Prevotella_9* | 0.020547945 | 0 | 0.020547945 | 125 |
| *Novosphingobium* | 0.020547945 | 0 | 0.020547945 | 125 |
| *Negativibacillus* | 0.020547945 | 0 | 0.020547945 | 125 |
| *Psychrobacter* | 0.01369863 | 0 | 0.01369863 | 130 |
| *Ruminiclostridium_6* | 0.01369863 | 0 | 0.01369863 | 130 |
| *Cloacibacterium* | 0.01369863 | 0 | 0.01369863 | 130 |
| *Mycobacterium* | 0.01369863 | 0 | 0.01369863 | 130 |
| *Cetobacterium* | 0.01369863 | 0 | 0.01369863 | 130 |
| *[Eubacterium]_coprostanoligenes* | 0.01369863 | 0 | 0.01369863 | 130 |
| *Holdemania* | 0.006849315 | 0 | 0.006849315 | 138 |
| *Streptococcus* | 0.006849315 | 0 | 0.006849315 | 138 |
| *Candidatus_Stoquefichus* | 0.006849315 | 0 | 0.006849315 | 138 |
| *Family_XIII_AD3011* | 0.006849315 | 0 | 0.006849315 | 138 |
| *Peptococcus* | 0.006849315 | 0 | 0.006849315 | 138 |
| *Coprobacter* | 0.006849315 | 0 | 0.006849315 | 138 |
| *Corynebacterium_1* | 0.006849315 | 0 | 0.006849315 | 138 |
| *Enterobacter* | 0.006849315 | 0 | 0.006849315 | 138 |
| *Fusobacterium* | 0.006849315 | 0 | 0.006849315 | 138 |
| *Lachnospiraceae_NC2004* | 0.006849315 | 0 | 0.006849315 | 138 |
| *Bacillus* | 0 | 0 | 0 | 145 |
| uncultured [f_ Christensenellaceae] | 0 | 0 | 0 | 145 |
| *Vitellibacter* | 0 | 0 | 0 | 145 |
| *Roseburia* | 0 | 0 | 0 | 145 |

This table presents the centrality metrics of bacterial genera identified from mucosal samples across four experimental groups: Control, AOM/DSS, AOM/DSS+DRB3 (defatted rice bran 3 g), and AOM/DSS+DRB6 (defatted rice bran 6 g). The following centrality measures were calculated to assess the influence of each genus within the microbial network: Degree Centrality: Indicates the number of direct interactions a genus has within the network, reflecting its importance as a microbial hub. Betweenness Centrality: Measures the extent to which a genus acts as a bridge in the network, influencing the flow of information between other taxa. Closeness Centrality: Reflects how efficiently a genus can spread interactions across the network. Rank: Genera are ranked in descending order based on their degree centrality, with a lower rank number indicating higher centrality and greater ecological influence.
